# A *Streptomyces* P450 enzyme dimerizes isoflavones from plants

**DOI:** 10.3762/bjoc.18.113

**Published:** 2022-08-26

**Authors:** Run-Zhou Liu, Shanchong Chen, Lihan Zhang

**Affiliations:** 1 Department of Chemistry, Fudan University, Shanghai 200433, China; 2 Key Laboratory of Precise Synthesis of Functional Molecules of Zhejiang Province, Department of Chemistry, School of Science, Westlake University, 18 Shilongshan Road, Hangzhou 310024, Zhejiang Province, China; 3 Institute of Natural Sciences, Westlake Institute for Advanced Study, 18 Shilongshan Road, Hangzhou 310024, Zhejiang Province, China

**Keywords:** biaryl coupling, cytochrome P450, dimerization, isoflavone, natural product

## Abstract

Dimerization is a widespread natural strategy that enables rapid structural diversification of natural products. However, our understanding of the dimerization enzymes involved in this biotransformation is still limited compared to the numerous reported dimeric natural products. Here, we report the characterization of three new isoflavone dimers from *Streptomyces cattleya* cultured on an isoflavone-containing agar plate. We further identified a cytochrome P450 monooxygenase, CYP158C1, which is able to catalyze the dimerization of isoflavones. CYP158C1 can also dimerize plant-derived polyketides, such as flavonoids and stilbenes. Our work represents a unique bacterial P450 that can dimerize plant polyphenols, which extends the insights into P450-mediated biaryl coupling reactions in biosynthesis.

## Introduction

Dimerization is a ubiquitous biotransformation in nature. Almost all forms of life, including bacteria, fungi, and plants, have the ability to produce homo- or heterodimeric natural products, which enables rapid structural diversification from simple monomers [1]. Often, the dimerized products exhibit significant biological activities due to the increased functional group density or complex stereochemistry, as seen in vinblastine [2], julichrome [3], himastatin [4], and biflavone [5]. However, the structural complexity of the dimeric natural products has hampered synthetic chemistry approaches towards these molecules, since chemo-, regio-, and atroposelective formation of the biaryl linkage remains highly challenging and often requires prefunctionalization of the substrate monomers, costly metal ligands, or tedious protection–deprotection steps [6–9].

With the advance of biosynthetic studies on natural products, a number of enzyme classes that are responsible for the dimer formation have been identified [1,10–14]. In plants and fungi, laccases and cytochrome P450 monooxygenases play pivotal roles in the biaryl bond formation of various polyketide dimers [10,15–16]. In contrast, in bacteria, P450 enzymes are the dominant catalysts, but no laccases have been reported for dimerization reactions (Figure S1, Supporting Information File 1). Due to the high reaction selectivity that the enzyme active site offers, these enzymes provide biocatalytic means for the biaryl linkage formation, and recent enzyme engineering efforts also demonstrated selective and efficient production of unnatural dimers or cross-coupling products, starting from simple monomers [17–19]. Nevertheless, our knowledge of enzyme-mediated dimerization is still limited in contrast to the numerous reported dimeric natural products.

Phenol coupling in plant polyphenol biosynthesis is one of the earliest documented biocatalytic dimerization reactions [20]. Contrary to flavone dimers and oligomers being abundant in nature, only limited dimeric compounds have been reported for isoflavones. Isoflavones bear a characteristic 3-phenylchroman skeleton, which is formed by the B-ring migration from the flavonoid scaffold catalyzed by isoflavone synthases [21]. The sporadic distribution of this isoflavone synthase limits the discovery of isoflavones in the plant kingdom [22], and the enzymes catalyzing isoflavone dimerization, to our knowledge, remain uncharacterized.

In this study, we report the discovery of a *Streptomyces* cytochrome P450 enzyme that catalyzes dimerization of plant isoflavones. By untargeted metabolomics, we isolated three new isoflavone dimers, namely cattleyaisoflavones A–C (**1**–**3**), from *Streptomyces cattleya* cultured in soy flour-containing media. We then identified a P450 enzyme, CYP158C1, which is able to dimerize the isoflavones as well as other plant-derived phenolic compounds. This work extends the insight on the substrate scope of P450-mediated dimerization and provides a potential biocatalytic tool for the synthesis of isoflavone dimers in future.

## Results and Discussion

### Characterization of three isoflavone dimers derived from daidzein (**4**)

**Figure 1:**
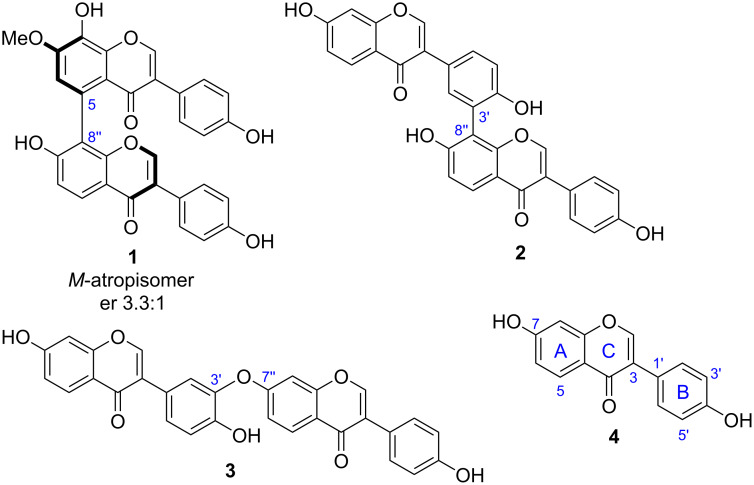
Structures of cattleyaisoflavones A (**1**), B (**2**), C (**3**), and daidzein (**4**).

During our effort of untargeted metabolomic screening of *Streptomyces* species, we identified several unknown isoflavone-like metabolites from *S. cattleya* NRRL8057 cultured on mannitol soya flour (MS) agar plates (Figure S2, Supporting Information File 1). Purification of the three major unknown compounds resulted in the isolation of the isoflavone dimers cattleyaisoflavones A (**1**), B (**2**), and C (**3**) (Figure 1). HRMS analyses suggested the molecular formula of both **2** and **3** to be C_30_H_18_O_8_ and that of **1** to be C_31_H_20_O_9_ (Figure S3, Supporting Information File 1), which matches the molecular formula of isoflavone dimers or dimeric derivatives.

**Figure 2:**
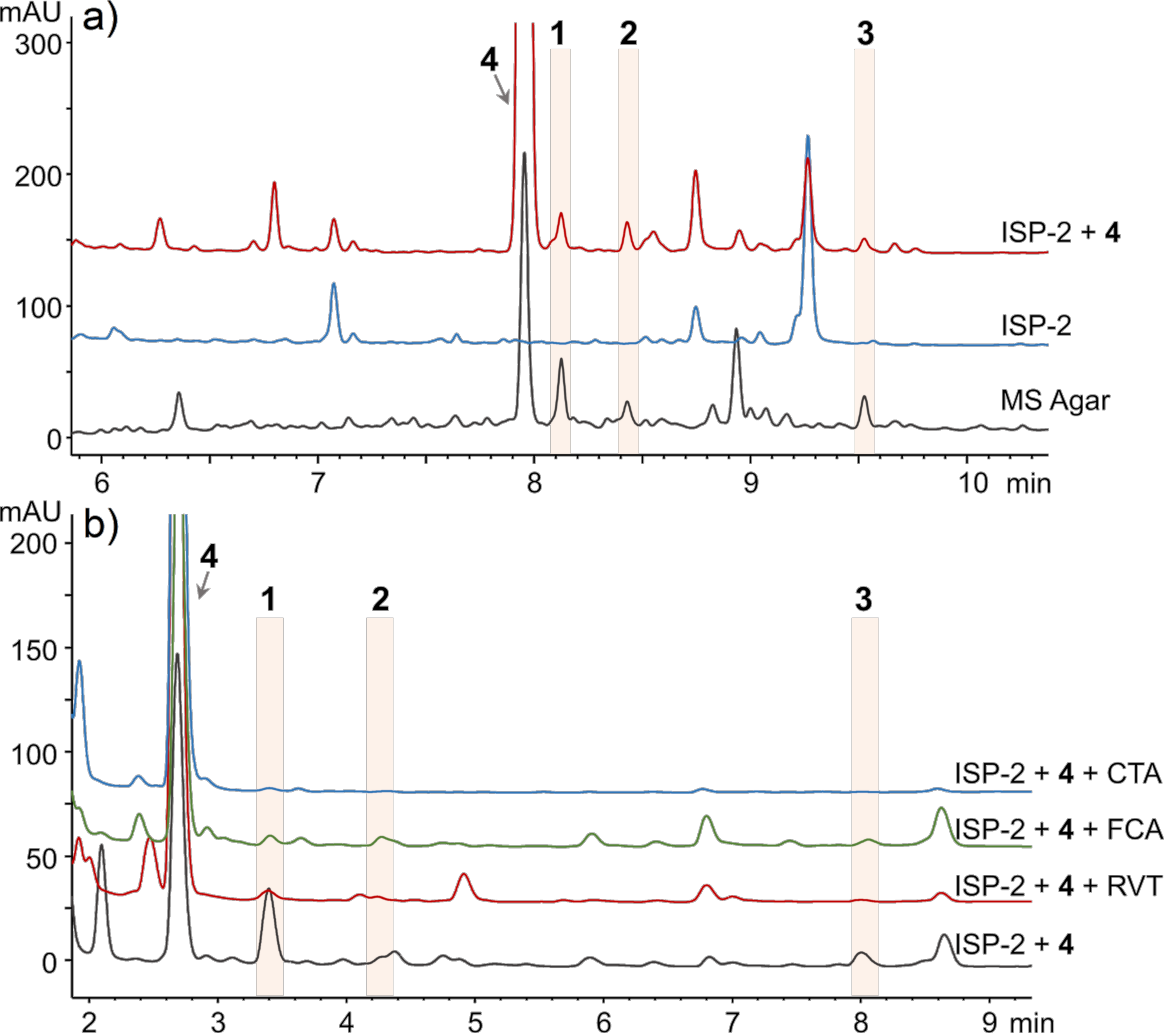
a) The culture in ISP-2 liquid did not produce **1**–**3**, while feeding with **4** restored the production (analytical method A according to Experimental section, 254 nm). b) Chemical genetics experiments indicated three P450 inhibitors could reduce the production of dimers derived from **4** (analytical method B to better analyze the isoflavone region, 254 nm). CTA: clotrimazole (2.5 µM), FCA: fluconazole (1000 µM), RVT: resveratrol (100 µM).

To confirm whether **1**–**3** were biosynthesized de novo by the strain or derived from the media that contain isoflavones, we cultured *S. cattleya* on International *Streptomyces* Project-2 (ISP-2) liquid medium that does not contain isoflavones. These cultures did not produce **1**–**3**, but daidzein (**4**) supplementation in ISP-2 liquid medium restored the production of **1**–**3**, suggesting that *S. cattleya* utilizes exogenous isoflavone to form the dimer products **1**–**3** (Figure 2a).

A comparison of the ^1^H NMR spectra of **1** and **4** (Tables S3 and S4, Supporting Information File 1) suggested that **1** has a biaryl linkage between the two A-rings. The ^1^H,^1^H-COSY correlation of H-5’’ (δ_H_ 8.06, d) and H-6’’ (δ_H_ 7.03, d) revealed C-8’’ as one of the coupling sites. The methoxy group (δ_H_ 3.98, s) was assigned to the C-7 site because of the ^1^H,^1^H-NOESY correlation with H-6 (δ_H_ 6.98, s). The HMBC correlations of H-6 to C-8 (δ_C_ 136.3), C-10 (δ_C_ 118.9), C-7 (δ_C_ 151.7), C-5 (δ_C_ 123.6), and C-8’’ (δ_C_ 119.1) suggested that C-5 is the coupling site on this unit, and C-8 was substituted by a hydroxy group. Finally, the structure of **1** was determined as a dimer with a rare 5,8’’ biaryl coupling skeleton. Likewise, C-8’’ was assigned as one of the coupling sites of **2**. Three B-ring protons, H-2’ (δ_H_ 7.38, d), H-5’ (δ_H_ 7.02, d), and H-6’ (δ_H_ 7.48, dd), suggested that C-3’ was another coupling site. Thus, the structure of **2** was established as a 3’,8’’-coupled dimer (Table S5, Supporting Information File 1). For the dimer **3**, three hydroxy and fifteen sp^2^ protons indicated a C–O bond linkage. An analysis of ^1^H,^1^H-COSY and HMBC spectra suggested it to be a 3’–O–7’’-coupled dimer (Table S6, Supporting Information File 1).

The absolute configuration of **1** and **2** with respect to atropisomerism was assessed by chiral HPLC analysis and the comparison of experimental and calculated ECD spectra. While **1** showed a ≈3:1 atropisomer ratio, with the *M*-atropisomer being the major form, **2** did not exhibit a Cotton effect in the ECD measurement, suggesting that it is in a freely rotating state (Supporting Information File 1).

### Discovery of the P450 enzyme responsible for dimerization

We next investigated which enzyme in *S. cattleya* is responsible for dimerization of **4**. The genome of *S. cattleya* (GCF_000237305.1) did not encode any laccase homologs but encoded 41 P450 enzymes in total. To investigate whether P450 enzymes mediate this dimerization reaction, we first employed a chemical genetics screening using three P450 inhibitors, CTA (strong inhibitor), FCA (moderate inhibitor), and RVT (polyphenol inhibitor) [23–24]. All three agents reduced the production of cattleyaisoflavones in a concentration-dependent manner (Figure 2b and Figure S4, Supporting Information File 1), suggesting that P450 enzymes play a key role in isoflavone dimerization.

**Figure 3:**
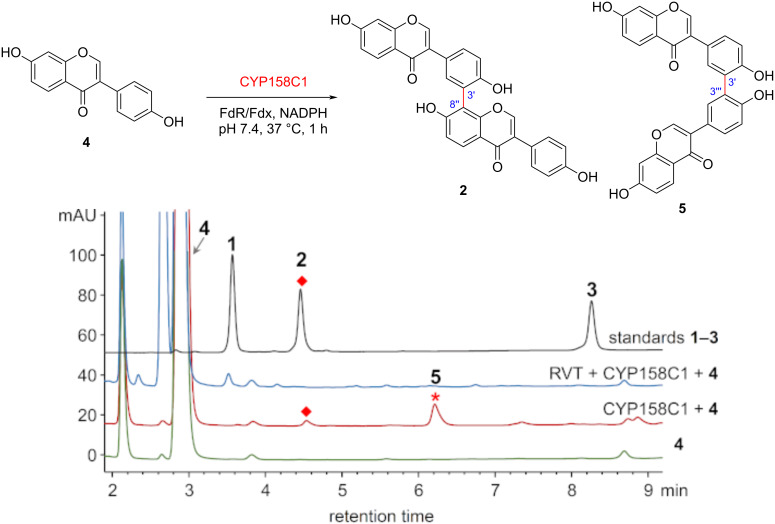
CYP158C1 dimerizes **4** to form dimers **2** and **5** in vitro (analytical method B, 254 nm). Control conditions (green): **4** (0.4 mM), NADPH (1 mM), and ferredoxin–NADP^+^ reductase *Sel*FdR0978/ferredoxin *Sel*Fdx1499 (FdR/Fdx, 5 µM) in buffer (50 mM PBS, 0.2 mM DTT, 1 mM EDTA, pH 7.4) were incubated at 37 °C for 1 h. Red: the conditions above with the addition of CYP158C1 (3 µM). Blue: the conditions above with the addition of CYP158C1 (3 µM) and RVT (0.5 mM).

The known bacterial P450 enzymes involved in biaryl coupling can be classified into four types, including CYP158 enzymes for dimerization of naphthols [25–27], JulI/SetI/SptI clade for anthraquinones [3], HmtS/ClpS/LtzS clade for cyclopeptides [4,18–19], and Bmp7 clade for polybrominated substrates (Table S7 and Figure S1, Supporting Information File 1) [13,17]. These P450 enzymes tend to have broad substrate specificity and form a variety of biaryl products. Thus, we constructed a phylogenetic tree using the 41 P450 enzymes in *S. cattleya*, together with these reported dimerization P450 enzymes (Figure S5, Supporting Information File 1). Notably, a P450 (WP_014145731.1, here termed CYP158C1) was clustered into the CYP158 clade that reportedly catalyzes dimerization of type III polyketide synthase (T3PKS) products, such as naphthols. Considering the similar biosynthetic pathway of isoflavones shared, this enzyme was expressed in *E. coli* and purified for in vitro biochemical assay together with four other P450 enzymes (CYP1–4) that were successfully expressed out of 10 P450 enzymes from each representative clade (Figures S5 and S6, Supporting Information File 1). The results showed that only CYP158C1 was able to dimerize **4** (Figure 3 and Figure S7, Supporting Information File 1), indicating that CYP158C1 is the most likely enzyme for isoflavone dimerization. Interestingly, different from the in vivo extract, in vitro experiments gave a major dimer **5**, a 3’,3’ coupled dimer assigned by MS–MS (Figures S8 and S14, Supporting Information File 1). Although dimer **3** was not visible in the HPLC analysis, a trace amount of **3** was detected by HRMS analysis (Figure S8, Supporting Information File 1). Redox partners might be responsible for the different regioselectivity between in vivo and in vitro reactions, as it has been reported that different reductases would lead to different diastereomeric dimers of coclaurine [28]. The absence of the product **1** in vitro is reasonable because the biosynthesis of **1** requires an additional methyltransferase from *S. cattleya* for the methoxy group formation.

### Substrate scope and reaction mechanism

**Scheme 1:**
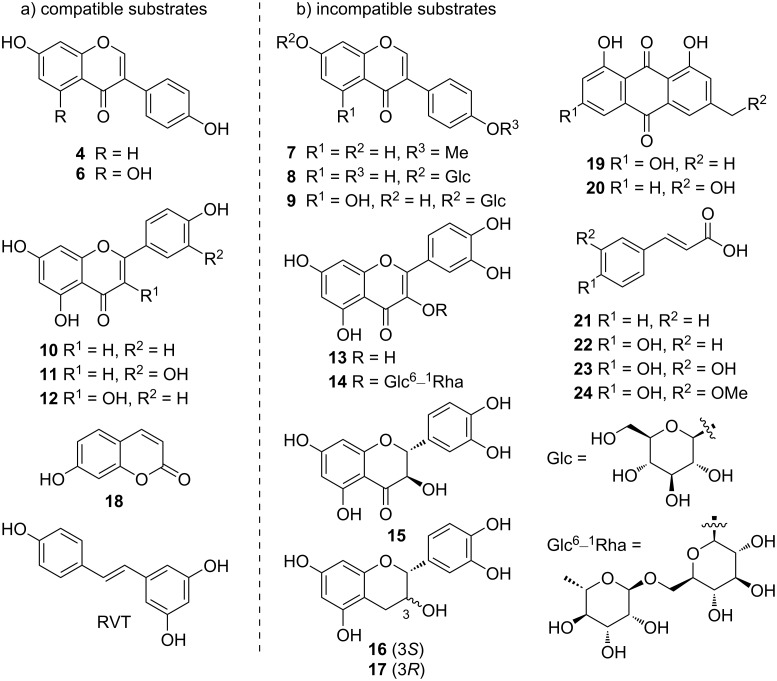
a) Compatible and b) incompatible substrates of CYP158C1. Products were identified using analytical method D.

After identification of CYP158C1 as the isoflavone dimerization enzyme, we next explored the substrate scope of the catalyst using a variety of plant-derived polyketides (Scheme 1). The results revealed that the 5-OH substitution on isoflavone (see compound **6**) was tolerable for dimerization (Figure S15, Supporting Information File 1), whereas the additional 4’-methoxyl group at the B-ring (see **7**) prevented the dimerization. Two isoflavone glucosides, **8** and **9**, failed to be transformed, presumably due to the excessive molecular size. When using the other flavonoids **10**–**17** as substrates, we observed the corresponding dimeric products for the substrates **10**–**12** (Figures S16–S18, Supporting Information File 1) but not for **13**–**17**, suggesting that CYP158C1 is also compatible with compounds with flavone scaffold. Apart from flavonoids, this enzyme also dimerized umbelliferone (**18**), a substrate of the fungal P450 KtnC [14,18], and RVT with relatively low efficiency (Figures S19 and S20, Supporting Information File 1). Other plant polyketides, such as anthraquinones **19** and **20** and phenylpropanoids **21**–**24**, failed to be dimerized.

**Scheme 2:**
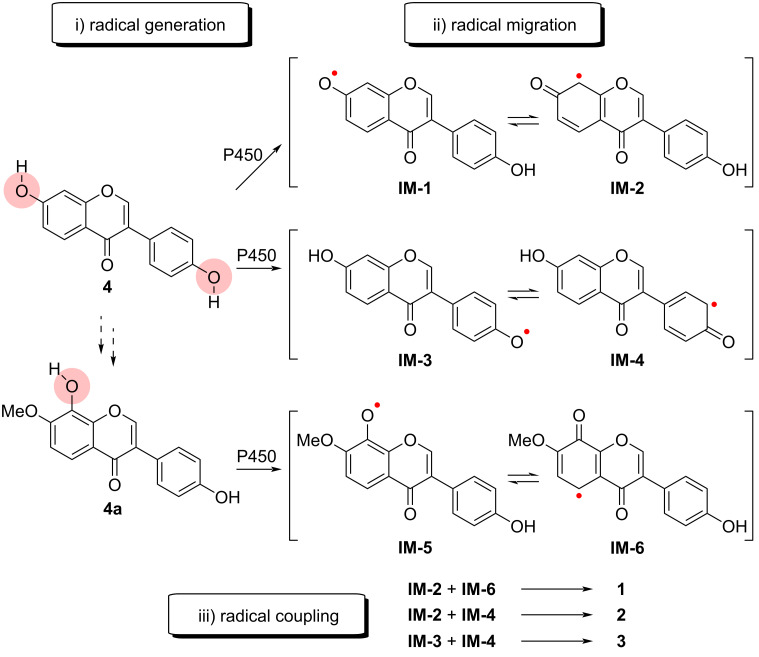
Proposed mechanism of CYP158C1-mediated dimerization of isoflavones.

The reaction mechanism of P450-mediated phenol dimerization is believed to involve oxidative radical–radical coupling, though other mechanisms, such as radical addition, radical cation addition, and electrophilic aromatic addition, have also been proposed [1,10,29]. A proposed mechanism is depicted in Scheme 2: First, the hydroxy group on the A- or B-ring is converted into a radical by a P450-induced single-electron transformation. The resulting radical then migrates to the π-system and is stabilized in the *ortho*- and *para*-positions, generating diverse carbon radical intermediates. As a result, various dimers are formed via promiscuous coupling of these radical intermediates.

Although we cannot exclude the possibility that other P450 enzymes in *S. cattleya* may also catalyze isoflavone dimerization, the above results indicated that CYP158C1 dimerizes isoflavones and several plant-derived aromatic polyketides, albeit with low catalytic efficiency. Future optimization of the reductase partner or reaction conditions may be required to improve the enzyme activity. Interestingly, we found that CYP158C1 clusters together with a T3PKS gene in the *S. cattleya* genome, which is similar to the biflaviolin [30] and naringenin [31] biosynthetic gene clusters. The native function of these type-III polyketide synthase products is believed to be involved in the protection of the host against oxidative radicals generated by UV irradiation [25,32]. To verify the antioxidative effect of the isoflavone dimers, 2,2'-azino-bis(3-ethylbenzothiazoline-6-sulfonic acid) (ABTS)-based antioxidant activity was performed [33]. The results showed that while **2** and **3** had an activity roughly equal to the monomers **4** and **6**, **1** displayed a roughly twofold radical scavenging capacity (Figure S9, Supporting Information File 1). This is in agreement with the previous report that the configuration of the hydroxy group of the B-ring plays a key role in the antioxidant activity [34]. Thus, CYP158C1 might provide an alternative pathway for antioxidant biosynthesis by using exogenous metabolites as substrates. The native substrate for CYP158C1, presumably produced by the clustered T3PKS, has yet to be identified.

The broad substrate specificity of CYP158C1 towards plant polyphenols suggests a potential host–microbial interaction in the rhizosphere. Flavones and isoflavones are common molecules in plant roots and can be secreted in soil, playing a role in allelopathic interference [35–36]. To our knowledge, this is the first bacterial P450 that can dimerize plant flavonoids. Since dimerization of polyphenols in plants is mostly catalyzed by laccases, the dimerization of (iso)flavones by a P450 in *Streptomyces* may represent a convergent evolution in enzyme chemistry between plants and bacteria.

## Conclusion

In conclusion, we have characterized three novel isoflavone dimers from *S. cattleya* and identified the P450 enzyme CYP158C1 to be able to catalyze biaryl coupling reactions using plant-derived daidzein (**4**) as a substrate. The enzyme can dimerize various flavonoids and provides a potential synthetic tool for the dimerization of aromatic polyketides.

## Experimental

### General experimental procedures

NMR spectra were acquired on Bruker 500 and 600 MHz AVANCE NEO spectrometers with TMS as an internal standard. HPLC–UV analysis was performed on an Agilent 1260 Infinity II HPLC system. UHPLC–HRMS analysis was performed on a Waters Synapt-G2-Si UHPLC-ESI-QToF-MS system. UV and CD spectra were acquired under N_2_ gas by a Chirascan V100 spectrometer (Applied Photophysics). Strain cultivation was performed at 28 °C in an incubator for agar plates or in a rotary shaker at 200 rpm for liquid media.

### Strain preparation and fermentation

The strain *S. cattleya* NRRL 8057 was purchased from the China General Microbiological Culture Collection Center (CGMCC). Seed culture of this strain was done in ISP-2 medium (yeast extract 0.4 g, malt extract 1 g, glucose 0.4 g, distilled water up to 100 mL, pH 7.2) for 2 days. Isoflavone dimer production was performed on MS agar medium (mannitol 20 g, soy flour 20 g, agar 20 g, distilled water up to 1 L) for 15 days from the above seed culture.

### Analytical methods

All HPLC analyses were performed using (A) H_2_O with 0.1% formic acid and (B) acetonitrile with 0.1% formic acid at 40 °C column temperature unless otherwise noted. The HPLC–UV analysis of feeding experiments was conducted with a YMC-Triart C18 ExRS column (150 mm × 3.0 mm, 3 μm, 12 nm, YMC) with a gradient of 5% B (0 min) to 95% B (12 min) at a flow rate of 0.5 mL/min (analytical method A). The HPLC–UV analysis of isoflavones was conducted with a Poroshell 120 SB-C18 column (100 mm × 3.0 mm, 2.7 μm, Agilent) with a gradient of 30% B (0 min) to 45% B (10 min) at a flow rate of 0.5 mL/min (analytical method B). The UHPLC–HRMS analysis of isoflavones was conducted with a HSS T3 column (100 mm × 2.1 mm, 1.8 μm, Waters) with a gradient of 30% B (0 min) to 45% B (4.5 min) at a flow rate of 0.4 mL/min (analytical method C). The UHPLC–HRMS analysis of the substrate scope was conducted with a HSS T3 column (100 mm × 2.1 mm, 1.8 μm, Waters) with a gradient of 0% B (0 min) to 100% B (5.0 min) at a flow rate of 0.4 mL/min (analytical method D). All HRMS analyses were performed in positive model with a scan range of *m*/*z* 50–1600. For MS–MS analysis, the collision-induced dissociation (CID) energy was set to 15–40 eV or 30–50 eV depending on the compounds. The HPLC–UV analysis of the chiral separation was conducted with a CHIRALCEL OX-3R column (150 mm × 4.6 mm, 3 μm, DAICEL) with isocratic 35% B at a flow rate of 1.0 mL/min at 30 °C column temperature, and an Agilent G6215B MSD detector was used for peak assignment (analytical method E).

### Isolation of compounds

To isolate compounds **1**–**3**, the fermentation culture of *S. cattleya* on MS agar medium (125 plates, 80 mL/plate each) was collected and extracted using organic solvent (CHCl_3_/MeOH 1:1, v/v, 20 L in total). After removing the solvent in vacuo, the crude extract was redissolved in methanol and separated by Sephdex LH-20 (GE healthcare) column chromatography using methanol as eluent. The yellow band at the elution tail on the LH-20 column (Figure S2, Supporting Information File 1) was collected and further purified by a Shimadzu LC-20AD semipreparative HPLC by using (A) H_2_O with 0.1% formic acid and (B) acetonitrile with 0.1% formic acid. Compounds **1** (5.0 mg), **2** (2.2 mg), and **3** (2.0 mg) were isolated using a YMC-Triart C18 column (250 mm × 10 mm, 5 μm, YMC) at 4 mL/min with a gradient of 40% B (0 min) to 60% B (12 min). A second round of purification was then performed for **1**–**3** with isocratic 35%, 38%, and 45% B, respectively, with a YMC-Triart Phenyl column (250 mm × 10 mm, 5 μm, YMC) at 4 mL/min.

**Cattleyaisoflavone A (1):** yellowish powder; UV (MeOH) λ_max_ (log ε) 257, 304 nm; for NMR spectral data, see Table S4, Supporting Information File 1; HRMS–ESI (*m*/*z*): [M + H]^+^ calcd for C_31_H_20_O_9_, 537.1180; found, 537.1206.

**Cattleyaisoflavone B (2):** yellowish powder; UV (MeOH) λ_max_ 255, 310 nm; for NMR spectral data, see Table S5, Supporting Information File 1; HRMS–ESI (*m*/*z*): [M + H]^+^ calcd for C_30_H_18_O_8_, 507.1074; found, 507.1091.

**Cattleyaisoflavone C (3):** white powder; UV (MeOH) λ_max_ 254, 300 nm; for NMR spectral data, see Table S6, Supporting Information File 1; HRMS–ESI (*m*/*z*): [M + NH^+^ calcd for C_30_H_18_O_8_, 507.1074; found, 507.1091.

### Feeding experiments

Feeding experiments were performed in 500 mL flasks each containing 100 mL of ISP-2 liquid medium and 10 mg daidzein (**4**, as 1 mL DMSO solution added after autoclave) by 1% inoculation from the above seed culture. On the 5th day, 2 mL of medium from each sample was taken and freeze-dried. Then, 1 mL MeOH was added and the mixture was treated with ultrasound for 15 min. After centrifugation, the supernatant was directly analyzed by HPLC following analytical method A.

### Chemical genetics experiments

All samples involved in inhibition experiments were set up using 100 mL flasks containing 20 mL ISP-2 medium, 0.02 mg/mL daidzein (**4**), and the P450 inhibitor, by 1% inoculation from the above seed culture. On the 9th day, 0.5 mL of medium from each sample was taken and freeze-dried. Then, 0.2 mL MeOH was added, and the mixture was treated with ultrasound for 15 min. After centrifugation, the supernatant was detected following analytical method B.

### Bioinformatic analysis

Multiple sequence alignment was constructed by MAFFT 7.450 using the E-INS-I method [37], and the phylogenetic tree was constructed by FastTree 2.1.11 [38] with default parameters by using Geneious Prime 2022.1.1 (see https://www.geneious.com).

### Protein purification and biochemical assays

The *Streptomyces* genome was extracted by TIANamp Bacteria DNA Kit (TIANGEN BIOTECH), and the P450 genes were amplified by PCR (Table S1, Supporting Information File 1) and cloned into a pET-28a(+) vector by the T5 exonuclease DNA assembly method [39]. The resulting expression vectors were individually introduced into *E. coli* BL21(DE3) for protein expression. Cells harboring expression plasmid were inoculated into LB medium with 50 µg/mL kanamycin and incubated at 37 °C until the culture reached an OD_600_ of 0.4–0.6. The cultures were then cooled to 16 °C and induced with 0.1 mM isopropyl β-ᴅ-1-thiogalactopyranoside (IPTG) for 16 h. Cells were harvested by centrifugation (10 min, 8,000 rpm, 4 °C).

For protein purification, cell pellets were resuspended in lysis buffer (50 mM NaH_2_PO_4_, 400 mM NaCl, 1 mM TCEP, pH 7.5) and lysed by sonication. After removing cell debris by centrifugation, the lysate supernatant was purified by Ni-NTA resin with washing buffer (50 mM NaH_2_PO_4_, 150 mM NaCl, 100 mM imidazole, pH 7.5) and elution buffer (50 mM NaH_2_PO_4_, 150 mM NaCl, 300 mM imidazole, pH 7.5) and then dialyzed into the storage buffer (50 mM NaH_2_PO_4_, 150 mM NaCl, 1 mM TCEP, 10% glycerol, pH 7.5) and stored at −80 °C for further use. The P450 partner proteins, FdR and Fdx, were constructed according to the reported method [40].

For in vitro biochemical assays, the reaction mixture (50 µL) contained 0.4 mM substrate, 3 µM P450 enzyme, 5 µM FdR, 5 µM Fdx, 1 mM NADPH, 0.2 mM DTT, and 1 mM EDTA in 50 mM PBS buffer (pH 7.4). The reaction mixture was incubated at 37 °C for 1 h and quenched by adding 50 µL MeOH. After centrifugation (10 min, 13,000 rpm), the mixture was subjected to HPLC–UV or UHPLC–HRMS analysis.

### Antioxidant activity

The antioxidant activity was assayed using a Total Antioxidant Capacity Assay Kit with ABTS method (Beyotime Biotechnology). Briefly, the fresh ABTS working solution was prepared by mixing ABTS stock solution with oxidant solution for an overnight reaction, and then this mixture was diluted 50 times by 80% ethanol. To initiate the assay reaction, 10 µL of each sample with a concentration of 1.0 mM in 80% ethanol was mixed with 200 µL of fresh ABTS working solution. After being incubated at 30 °C for 5 min, the 734 nm absorbance was measured on a Thermo Varioskan LUX Microplate reader. Trolox was used as the reference compound for calculation of ABTS-reducing activity (in %).

### Computational ECD calculation of compound **1**

Merck molecular force field (MMFF) and DFT as well as TDDFT calculations were carried out with the Spartan 14 software (Wavefunction Inc.) and the Gaussian 16 program [41], respectively. Conformers within the 2 kJ/mol energy window were generated and optimized using DFT calculations at the B3LYP/6-31+G (d,p) level. Frequency calculations were performed at the same level to confirm that each optimized conformer was true minimum and to estimate the relative thermal free energy (Δ*G*) at 298.15 K. The two conformers were chosen for ECD calculations in methanol at the B3LYP/6-311+G (d,p) level. Solvent effects were taken into consideration using the self-consistent reaction field (SCRF) method with the polarizable continuum model (PCM). The ECD spectrum was generated by the SpecDis program [42] using a Gaussian band shape with 0.23 eV exponential half-width from dipole length rotational strengths.

## Supporting Information

File 1 Detailed descriptions of the experimental procedures and comprehensive analytical data.
