# Supplementary material for: A Streptomyces P450 enzyme dimerizes isoflavones from plants
Source: Beilstein J Org Chem. 2022 Aug 26;18:1107–15. doi: 10.3762/bjoc.18.113 (PMC9443421; doi:10.3762/bjoc.18.113)
Supplement: File 1 — Detailed descriptions of the experimental procedures and comprehensive analytical data. [file Beilstein_J_Org_Chem-18-1107-s001.pdf]

BEILSTEIN JOURNAL OF ORGANIC CHEMISTRY

Supporting Information

for

A *Streptomyces* P450 enzyme dimerizes isoflavones from plants

Run-Zhou Liu, Shanchong Chen and Lihan Zhang

Beilstein J. Org. Chem. **2022,** *18,* 1107–1115. doi:10.3762/bjoc.18.113

Detailed descriptions of the experimental procedures and comprehensive analytical data

License and Terms: This is a supporting information file under the terms of the Creative Commons Attribution License (https://creativecommons.org/licenses/by/4.0). Please note that the reuse, redistribution and reproduction in particular requires that the author(s) and source are credited and that individual graphics may be subject to special legal provisions.
The license is subject to the *Beilstein Journal of Organic Chemistry* terms and conditions: (https://www.beilstein-journals.org/bjoc/terms)

Table of contents

1. Supplementary tables S**2**

Table S1. Primers used in this study S2

Table S2. Substrates and P450 inhibitors used in this study S2

Table S3. NMR signal assignments for **4** in MeOH-d_4 and DMSO-d_6 .... S3

Table S4. NMR signal assignments for **1** in MeOH-d_4 .... S4

Table S5. NMR signal assignments for **2** in MeOH-d_4 .... S5

Table S6. NMR signal assignments for **3** in DMSO-d_6 .... S6

Table S7. Bacterial P450 enzymes involved in the biaryl coupling of dimeric NPs S7

2. Supplementary figures part I S**8**

Figure S1. Summary of known bacterial P450 enzymes for biaryl coupling S8

Figure S2. Preparation and analysis of the isoflavone-contained fraction S10

Figure S3. HRMS spectra of purified compounds **1**–**3** S10

Figure S4. Spectra of chemical genetics experiments S11

Figure S5. The phylogenetic tree analysis S11

Figure S6. SDS-PAGE analysis of the purified proteins S12

Figure S7. Biochemical assays in vitro S12

Figure S8. Comparison of dimers derived from **4** in vivo and in vitro by HRMS analysis S13

Figure S9. The antioxidant activity of **1**–**4** and **6** S13

3. Supplementary figures part II: MS–MS analyses for dimeric products S**14**

Figures S10–S14. The MS–MS analysis of compounds **1**–**6** S14

Figures S15–S20. The MS–MS analysis of dimers from substrate scope investigation S19

4. Supplementary figures part III: data of compounds 1–**3** S**28**

Figures S21–S26. NMR spectra of compound **1**. S28

Figures S27–S30. NMR spectra of compound **2** S31

Figures S31–S34. NMR spectra of compound **3** S34

Figure S35. UV and CD spectra of **1**–**3** S36

Figure S36. The HPLC analyses of **1** and **2** using chiral column S37

Figures S37 and S38. Experimental and calculated ECD spectra of **1** S38

5. References S**39**

1. Supplementary tables

Table S1. Primers used in this study.

target protein	primer	sequence (5'→3')
WP_014628006	CYP1-F	CGCGGCAGCCATATGATGAGCGACCCGACGACAGAC
(CYP1)	CYP1-R	GTGGTGCTCGAGTCACTAGGTAAGAACCGGCAACGCCGC
WP_014143996	CYP2-F	CGCGGCAGCCATATGATGAGCAAGCAGGCGCCGTTG
(CYP2)	CYP2-R	GTGGTGCTCGAGTCATCACGCCGCGGCCTCCCCGCT
WP_014144304	CYP3-F	CGCGGCAGCCATATGGTGTCCGTGACCCGACCCTCG
(CYP3)	CYP3-R	GTGGTGCTCGAGTCATCAGTCCAGCAGCACCGGCAG
WP_014146459	CYP5-F	CGCGGCAGCCATATGATGACAAGTGCGAACAACGACGCC
(CYP4)	CYP5-R	GTGGTGCTCGAGTCATCAGTCGCGAGGGCCGGGCCC
WP_014145731	158C1-F	CGCGGCAGCCATATGATGACCACCCGACCCCACGAG
(CYP158C1)	158C1-R	GTGGTGCTCGAGTCATCACCAGGTCACCGGGAGCGC

Table S2. Substrates and P450 inhibitors used in this study. [a]

compound	trivial name	CAS No.	formula	MW	purity
**4**	daidzein	486-66-8	$C_{15}H_{10}O_4$	254.24	98%
**6**	genistein	446-72-0	$C_{15}H_{10}O_5$	270.24	98%
**7**	formononetin	485-72-3	$C_{16}H_{12}O_4$	268.26	98%
**8**	daidzin	552-66-9	$C_{21}H_{20}O_9$	416.40	95%
**9**	genistin	529-59-9	$C_{21}H_{20}O_{10}$	432.40	98%
**10**	apigenin	520-36-5	$C_{15}H_{10}O_5$	270.24	98%
**11**	luteolin	491-70-3	$C_{15}H_{10}O_6$	286.24	98%
**12**	kaempferol	520-18-3	$C_{15}H_{10}O_6$	286.24	98%
**13**	quercetin	117-39-5	$C_{15}H_{10}O_7$	302.23	97%
**14**	rutin	153-18-4	$C_{27}H_{30}O_{16}$	610.50	98%
**15**	taxifolin	480-18-2	$C_{15}H_{12}O_7$	304.25	98%
**16**	(+)-catechin	154-23-4	$C_{15}H_{14}O_6$	290.27	97%
**17**	(−)-epicatechin	490-46-0	$C_{15}H_{14}O_6$	290.27	97%
**18**	umbelliferone	93-35-6	$C_9H_6O_3$	162.14	98%
**19**	emodin	518-82-1	$C_{15}H_{10}O_5$	270.24	98%
**20**	aloe emodin	481-72-1	$C_{15}H_{10}O_5$	270.24	97%
**21**	cinnamic acid	621-82-9	$C_9H_8O_2$	148.16	99%
**22**	coumaric acid	501-98-4	$C_9H_8O_3$	164.16	98%
**23**	caffeic acid	331-39-5	$C_9H_8O_4$	180.16	98%
**24**	ferulic acid	1135-24-6	$C_{10}H_{10}O_4$	194.18	98%
CTA	clotrimazole	23593-75-1	$C_{22}H_{17}ClN_2$	344.80	99%
FCA	fluconazole	86386-73-4	$C_{13}H_{12}F_2N_6O$	306.27	98%
RVT	resveratrol	501-36-0	$C_{14}H_{12}O_3$	228.24	99%

[a]All compounds were commercially available from Bide Pharmatech, Macklin Biochemical or Energy Chemical.

Table S3. NMR signal assignment for **4** in MeOH-d_4 and DMSO-d_6.[a]

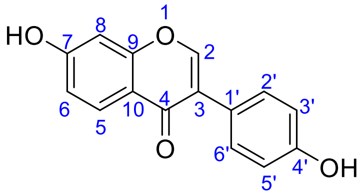

No	daidzein (**4**) in MeOH-d_4		daidzein (**4**) in DMSO-d_6	
	δ_H (J in Hz)	δ_C	δ_H (J in Hz)	δ_C
2	8.13 (s)	154.7	8.29 (s)	152.8
3		124.3		123.5
4		178.2		174.7
5	8.05 (d, 8.8)	128.5	7.97 (d, 8.8)	127.3
6	6.93 (dd, 8.8, 2.3)	116.5	6.94 (dd, 8.8, 2.3)	115.1
7		165.1		162.5
8	6.85 (d, 2.3)	103.2	6.87 (d, 2.3)	102.1
9		158.7		157.4
10		118.2		116.6
1’		126.0		122.6
2’	7.36 (d, 8.6)	131.4	7.39 (d, 8.6)	130.1
3’	6.84 (d, 8.6)	116.2	6.81 (d, 8.6)	115.0
4’		159.8		157.2
5’	6.84 (d, 8.6)	116.2	6.81 (d, 8.6)	115.0
6’	7.36 (d, 8.6)	131.4	7.39 (d, 8.6)	130.1
7-OH			10.78 (br s)	
4’-OH			9.53 (br s)	

[a]The ^1H and ^{13}C NMR spectra of daidzein (**4**) were acquired using commercially available standard (purity 98%) [1,2].

Table S4. NMR signal assignment for **1** in MeOH-d_4.

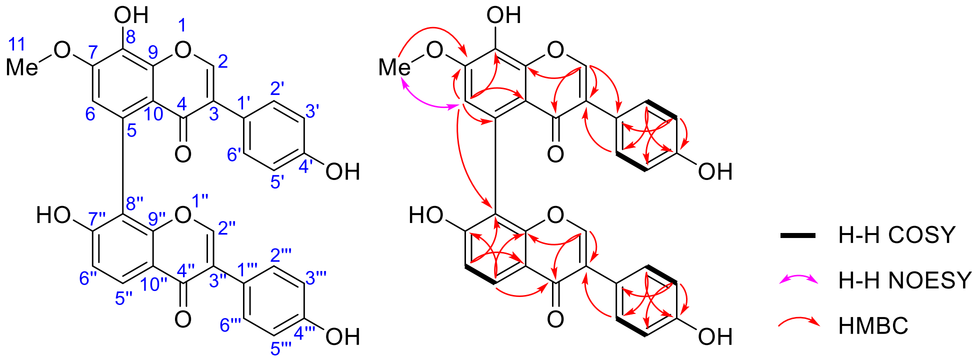

	cattleyaisoflavone A (**1**)			
No	δ_H (*J* in Hz)	δ_C	COSY	HMBC
2	8.16 (s)	153.7, CH		3, 4, 9, 1’
3		126.2, C		
4		178.7, C		
5		123.6, C		
6	6.98 (s)	114.9, CH		5, 7, 8, 10, 8”
7		151.7, C		
8		136.3, C		
9		148.6, C		
10		118.9, C		
11	3.98 (s)	57.2, CH_3		7
1’		124.3, C		
2’	7.22 (d, 8.6)	131.5, CH	3’	3, 6’, 4’
3’	6.75 (d, 8.6)	116.2, CH	2’	1’, 5’, 4’
4’		158.6, C		
5’	6.75 (d, 8.6)	116.2, CH	6’	1’, 3’, 4’
6’	7.22 (d, 8.6)	131.5, CH	5’	3, 2’, 4’
2”	7.96, (s)	154.7, CH		3”, 4”, 9”
3”		125.6, C		
4”		178.8, C		
5”	8.06 (d, 8.9)	126.5, CH	6”	4”, 7”, 9”
6”	7.03 (d, 8.9)	115.9, CH	5”	8”, 10”
7”		160.8, C		
8”		119.1, C		
9”		157.3, C		
10”		118.3, C		
1’”		124.5, C		
2’”	7.34 (d, 8.6)	131.5, CH	3’”	3”, 6’”, 4’”
3’”	6.81 (d, 8.6)	116.2, CH	2’”	1’”, 5’”, 4’”
4’”		158.6, C		
5’”	6.81 (d, 8.6)	116.2, CH	6’”	1’”, 3’”, 4’”
6’”	7.34 (d, 8.6)	131.5, CH	5’”	3”, 2’”, 4’”

Table S5. NMR signal assignment for **2** in MeOH-d_4.

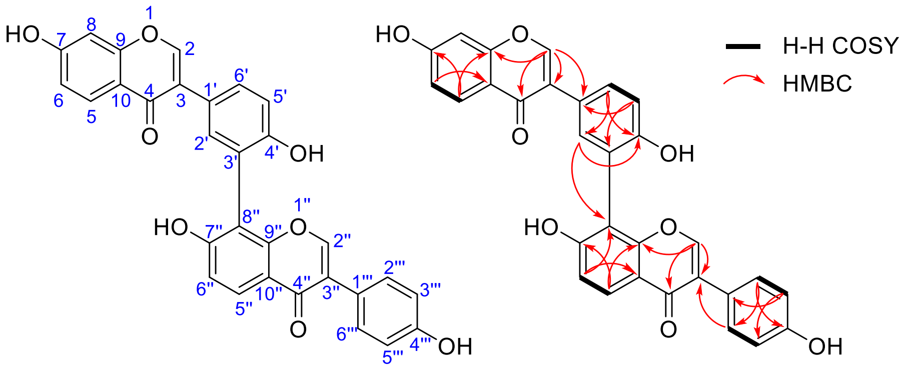

cattleyaisoflavone B (**2**)				
No	δH (*J* in Hz)	δC [a]	COSY	HMBC
2	8.20 (s)	154.9, CH		3, 4, 9, 1’
3		125.9, C		
4		178.4, C		
5	8.06 (d, 8.8)	128.7, CH	6	7, 9
6	6.94 (d, 8.8, 2.3)	116.6, CH	5	10
7		164.8, C		
8	6.86 (d, 2.3)	103.4, CH		
9		159.9, C		
10		118.2, C		
1’		124.5, C		
2’	7.38 (d, 2.3)	134.3, CH		4’, 8”
3’		120.3, C		
4’		157.0, C		
5’	7.02 (d, 8.5)	116.9, CH	6’	1’, 3’
6’	7.48 (d, 8.5, 2.3)	131.8, CH	5’	2’, 4’
2”	8.06 (s)	155.0, CH		3”, 4”, 9”
3”		125.6, C		
4”		178.8, C		
5”	8.11 (d, 8.9)	127.3, CH	6”	7”, 9”
6”	7.08 (d, 8.9)	116.3, CH	5”	8”, 10”
7”		161.9, C		
8”		114.8, C		
9”		157.7, C		
10”		118.5, C		
1”’		124.5, C		
2”’	7.36 (d, 8.6)	131.5, CH	3”’	3”, 4”’, 6”’
3”’	6.83 (d, 8.6)	116.3, CH	2”’	1”’, 5”’
4”’		158.8, C		
5”’	6.83 (d, 8.6)	116.3, CH	6”’	1”’, 3”’
6”’	7.36 (d, 8.6)	131.5, CH	5”’	3”, 2”’, 6”’

[a]Observed by HSQC or HMBC.

Table S6. NMR signal assignment for **3** in DMSO-d_6.

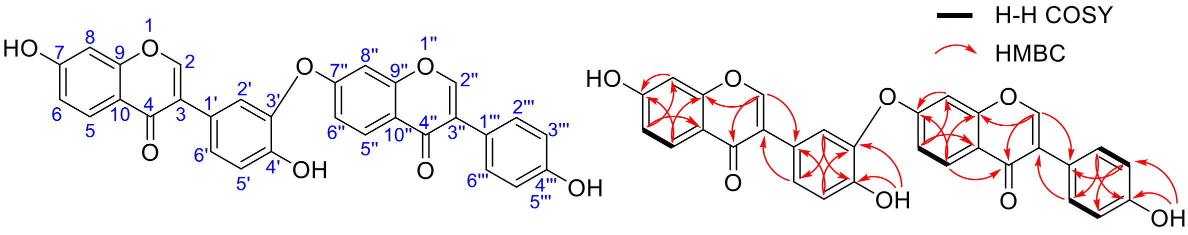

cattleyaisoflavone C (3)				
No	δH (*J* in Hz)	δC [a]	COSY	HMBC
2	8.36 (s)	153.8		4, 9, 1’
3		123.8		
4		175.3		
5	8.10 (d, 8.9)	127.8	6	7, 9
6	7.09 (dd, 8.9, 2.4)	115.2	5	8, 10
7		163.0		
8	6.94 (d, 2.4)	103.6		6, 7, 9, 10
9		157.2		
10		118.8		
1’		124.1		
2’	7.45 (d, 2.1)	122.8		4’, 6’
3’		140.9		
4’		149.4		
5’	7.09 (d, 8.4)	117.0	6’	1’, 3’
6’	7.41 (dd, 8.4, 2.1)	127.7	5’	3
7-OH	10.81 (br s)			
4’-OH	9.99 (br s)			3’, 4’
2”	8.42 (s)	153.8		4”, 9”, 1’”
3”		123.8		
4”		174.9		
5”	7.96 (d, 8.8)	127.8	6”	4”, 7”, 9”
6”	6.94 (dd, 8.8, 2.2)	115.7	5”	8”, 10”
7”		163.5		
8”	6.87 (d, 2.2)	102.8		6”, 7”, 9”, 10”
9”		157.8		
10”		117.2		
1’”		123.0		
2’”	7.40 (d, 8.6)	130.1	3’”	3”, 4’”, 6’”
3’”	6.81 (d, 8.6)	115.3	2’”	1’”, 5’”
4’”		157.8		
5’”	6.81 (d, 8.6)	115.3	6’”	1’”, 3’”
6’”	7.40 (d, 8.6)	130.1	5’”	3”, 2’”, 6’”
4’”-OH	9.55 (br s)			3’”, 4’”

[a]Observed by HSQC or HMBC.

Table S7. Bacterial P450 enzymes involved in the biaryl coupling of dimeric NPs.

enzyme	organism	substrate type (exp.)	biosynthetic source	Reference
P450mel[a]	*S. griseus*	naphthalene	type III PKS	[3]
CYP158A1	*S. coelicolor*	naphthoquinone	type III PKS	[4]
CYP158A2	*S. coelicolor*	naphthoquinone	type III PKS	[5]
CYP158A3	*S. avermitilis*	naphthoquinone[b,c]	type III PKS	[6]
JulI	*S. afghaniensis*	anthraquinone	type II PKS	[7]
SetI	*S. aurantiacus*	(seq.)		[7]
SptI	*S. spectabilis*	(seq.)		[7]
HmtS	*S. himastatinicus*	cyclopeptides[c]	NRPS	[8,9]
ClpS	*S. sp.* MK498-98 F14	cyclopeptides[b,c]	NRPS	[9]
LtzS	*L. flaviverrucosa*	cyclopeptides	NRPS	[10]
Bmp7	*P. luteoviolacea*	polybrominated NPs[c]	others	[11,12]
Pp_Bmp7	*P. phenolica*	(seq.)		[11]
Mm_Bmp7	*M. mediterranea*	(seq.)		[11]

[a]P450mel is also called CYP158B1 [13]. [b]The function of this enzyme was not verified using native substrate. [c]These enzymes can dimerize nonnative substrates as Figure S1 shows.

2. Supplementary figures part I

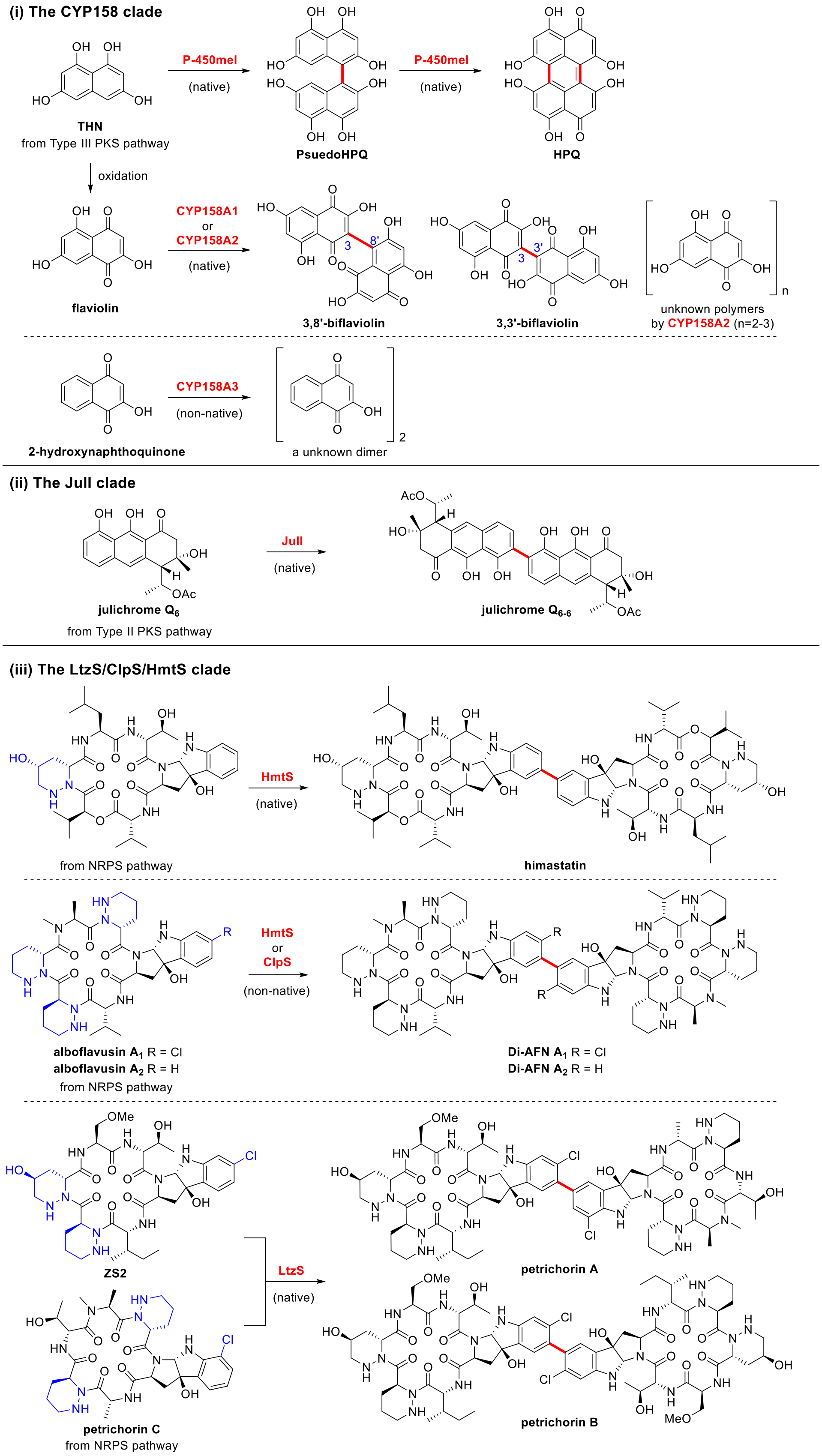

Figure S1. Summary of known bacterial P450 enzymes for biaryl coupling.

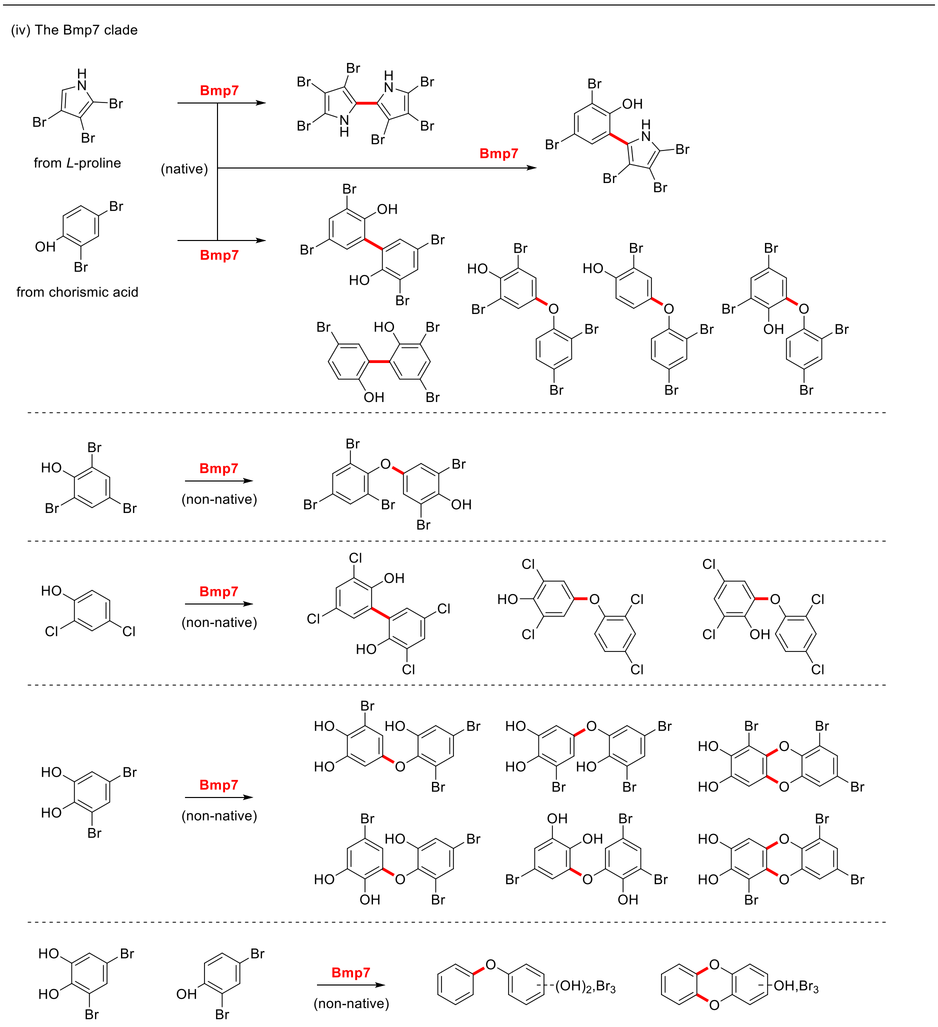

Figure S1. Summary of known bacterial P450 enzymes for biaryl coupling (continued).

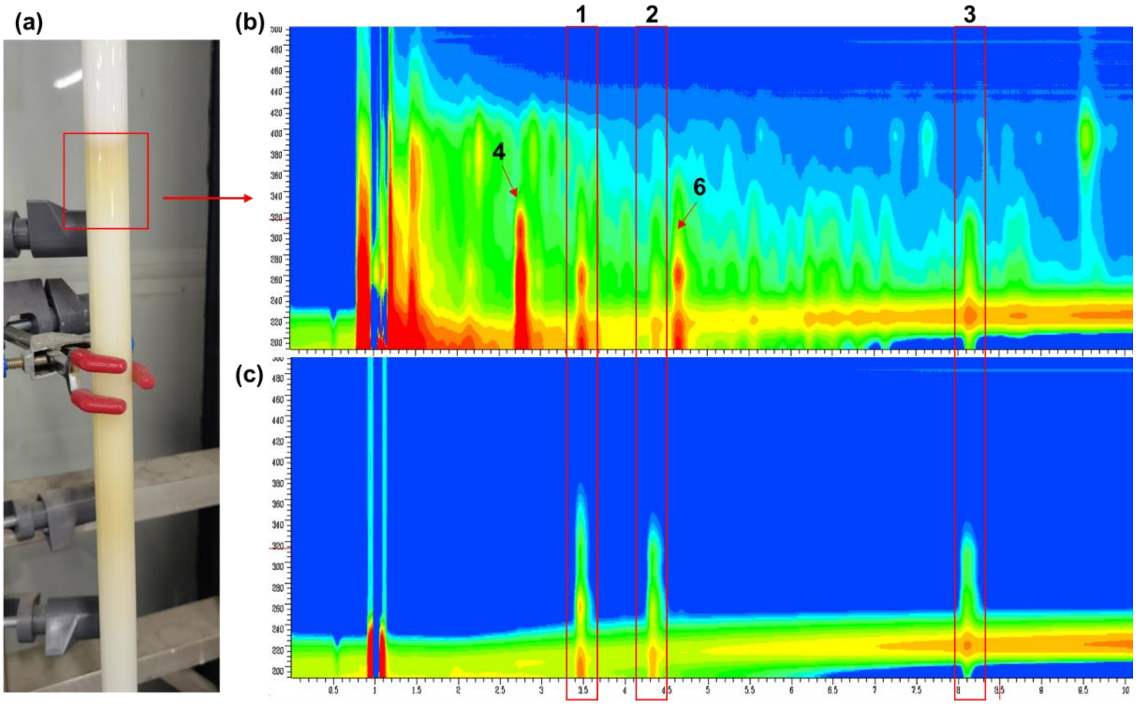

Figure S2. Preparation and analysis of the isoflavone-contained fraction. (a) When separating the extract of *S. cattleya* cultivated using MS agar plates, the yellow color band at the elution tail on Sephadex LH-20 column was collected. The 3D UV spectra of (b) this fraction and (c) isolated compounds **1**–**3** were acquired following analytical method B.

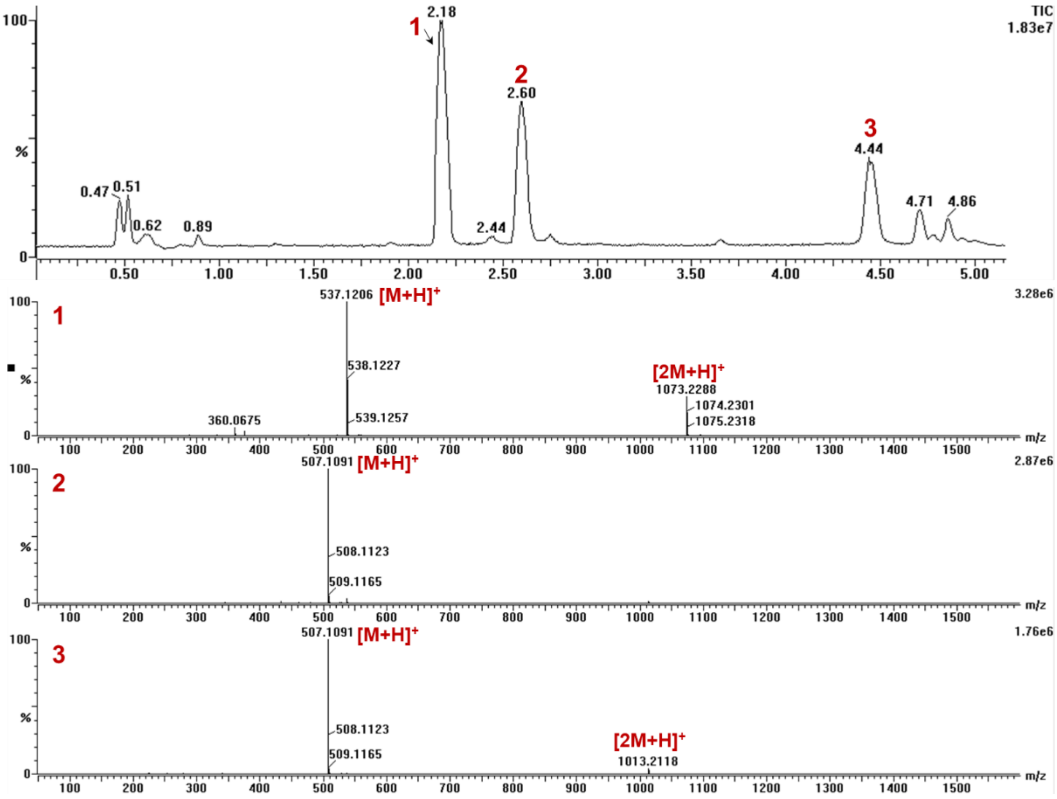

Figure S3. HRMS spectra of purified compounds **1**–**3** (analytical method C).

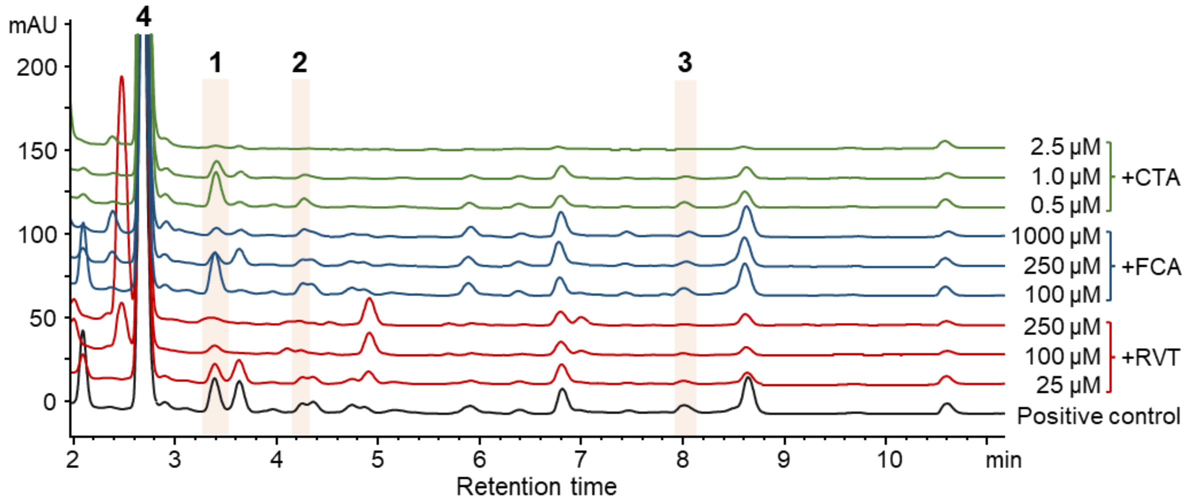

Figure S4. Spectra of chemical genetics experiments using three P450 inhibitors (analytical method B, 254 nm).

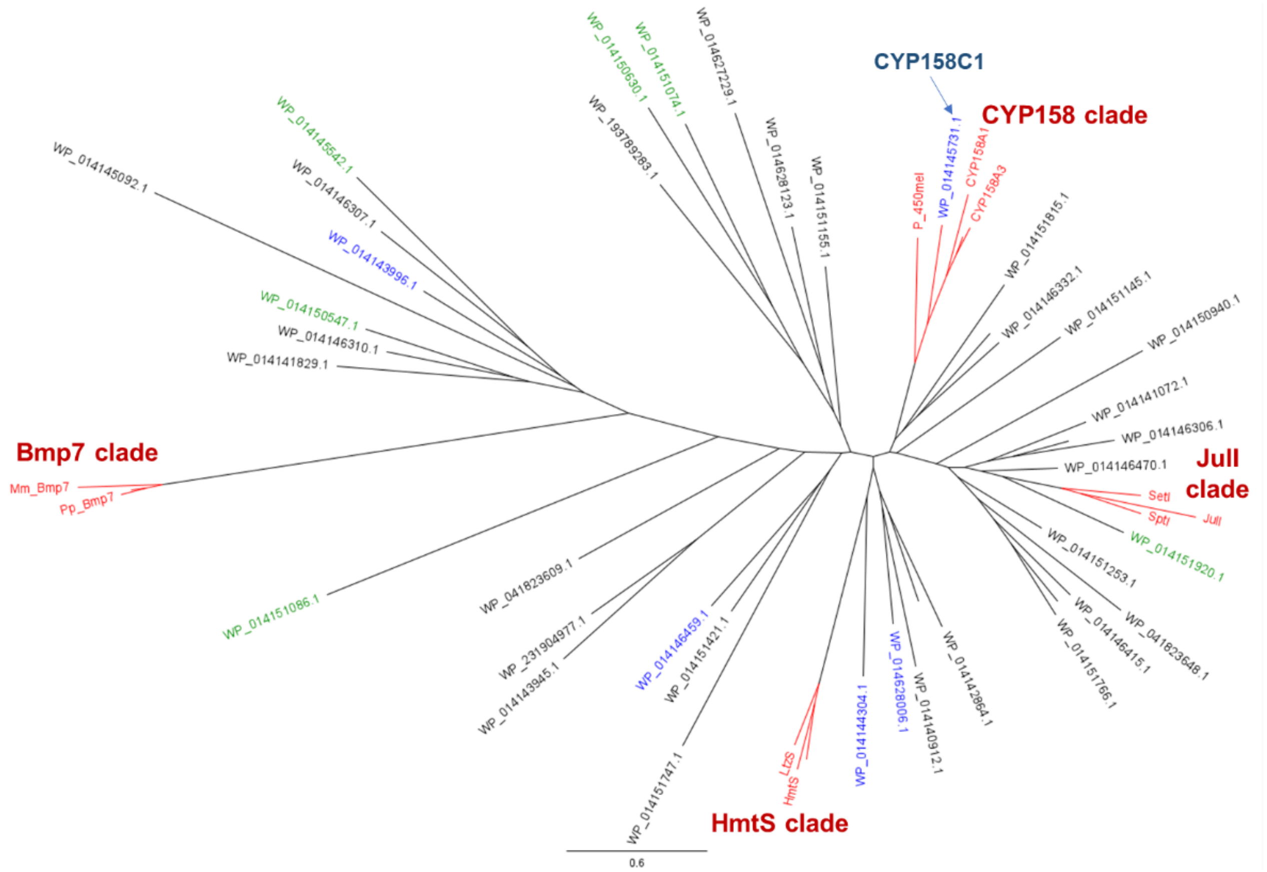

Figure S5. The phylogenetic tree of 41 P450s in *S. cattleya* and 13 known bacterial P450s involved in biaryl coupling. 13 proteins in red: known bacterial P450s summarized in Table S7 and Figure S1. CYP158C1 together with 10 other P450s that show close distance to known biaryl coupling P450s or from major clades were selected from this tree for screening. As a result, 5 proteins in blue were successfully expressed and purified, including CYP1, WP_014628006.1; CYP2, WP_014143996.1; CYP3, WP_014144304.1; CYP4, WP_014146459.1; and CYP158C1, WP_014145731.1 (Figure S6). The green label indicates P450s which we were not able to obtain soluble proteins.

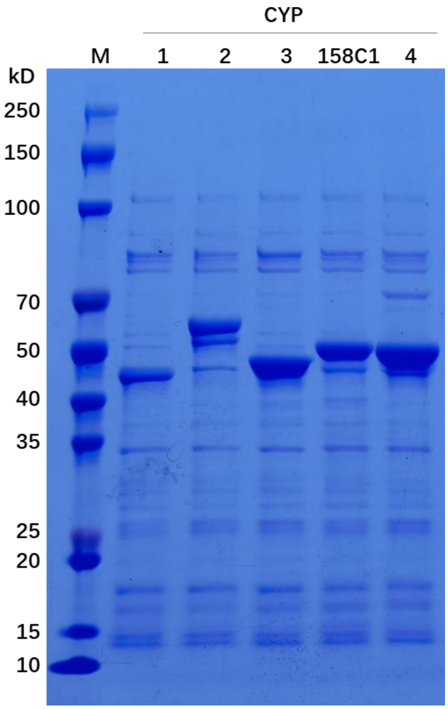

Figure S6. SDS-PAGE analysis of P450 enzymes purified in this study. His6-CYP1 (44.8 kD), His6-CYP2 (52.4 kD), His6-CYP3 (47.1 kD), His6-CYP158C1 (48.7 kD), and His6-CYP4 (48.6 kD). M: marker.

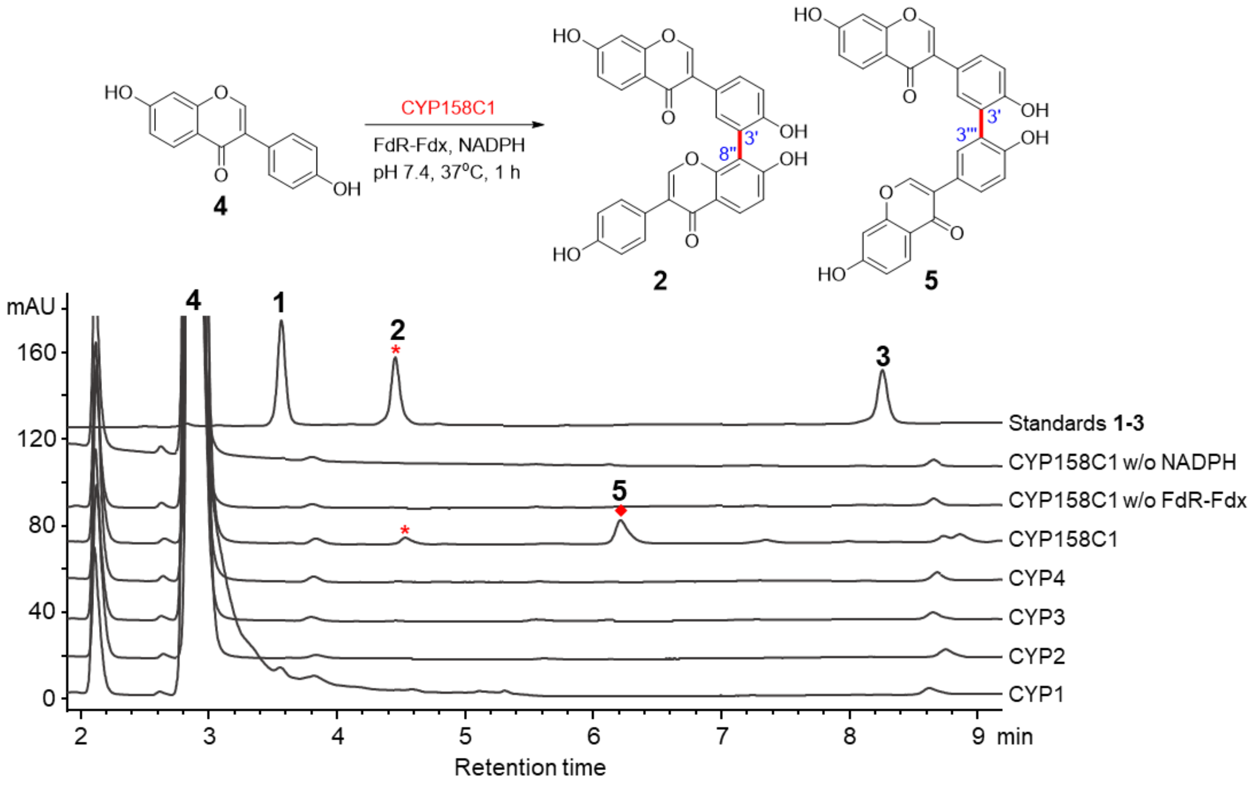

Figure S7. Biochemical assays in vitro using substrate **4**. The reaction methods followed experimental part (analytical method B, 254 nm).

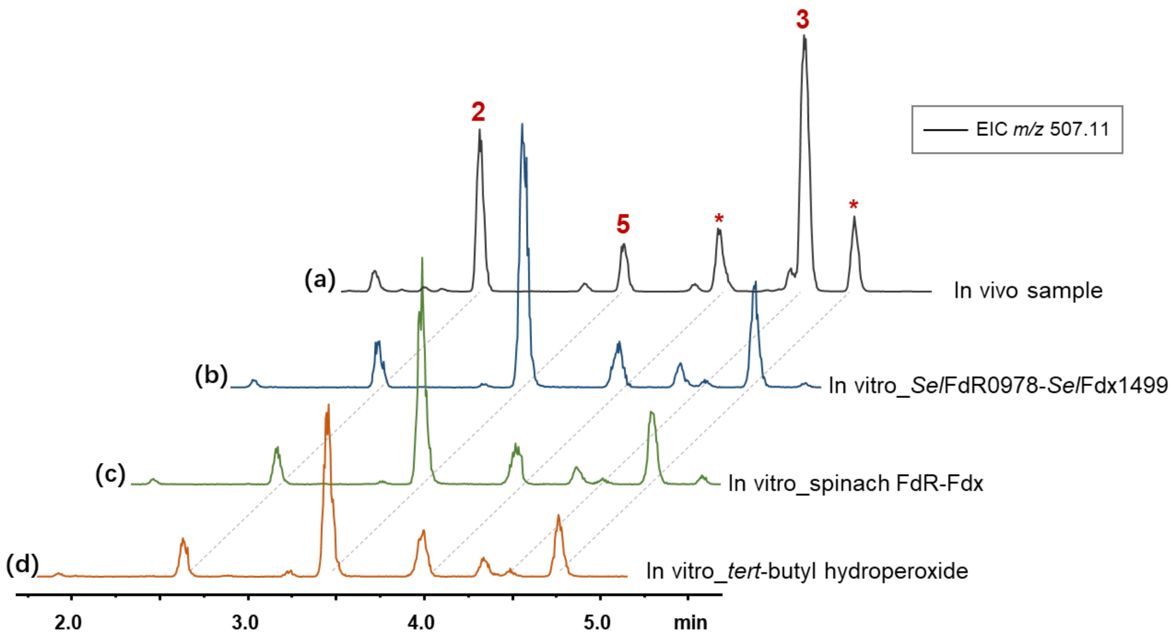

Figure S8. Comparison of dimers derived from **4** in vivo and in vitro by HRMS analysis. (a) The sample in vivo was the isoflavone-contained fraction used in Figure S2. For the samples in vitro, different redox partners were tested following the method described in Experimental part, including (b) *SeI*FdR0978/*SeI*Fdx1499 used in main text, (c) spinach FdR/Fdx used in reference [3] and (d) *tert*-butyl hydroperoxide used in reference [6]. UHPLC–(+)HRMS–ESI analysis followed the analytical method C. Asterisks (*) represent uncharacterized dimers. The spinach FdR and Fdx were purchased from Sigma-Aldrich, and *tert*-butyl hydroperoxide was from Energy Chemical.

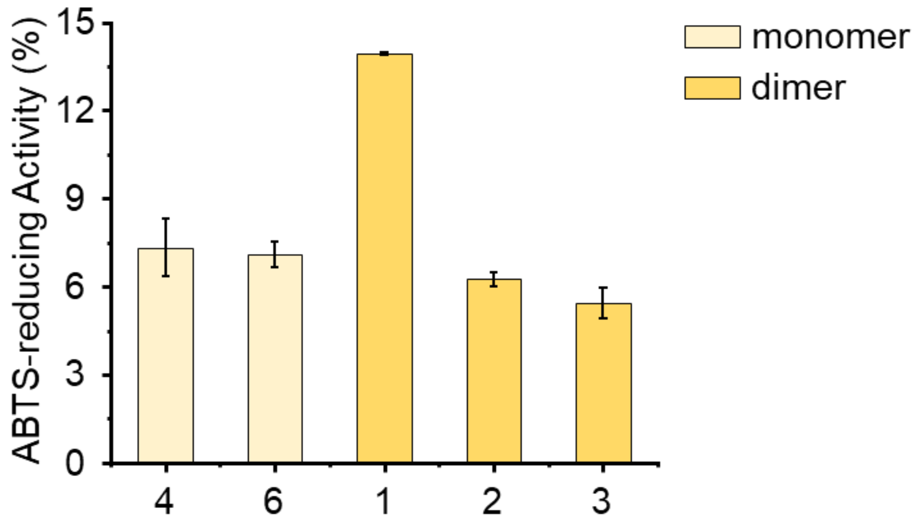

Figure S9. The antioxidant activity of daidzein (**4**), genistein (**6**), and isoflavone dimers **1**–**3** (n = 3).

3. Supplementary figures part II: MS–MS analyses for dimeric products

Figure S10. The MS–MS analysis of daidzein (**4**) and genistein (**6**). UHPLC–(+)HRMS–ESI analysis followed analytical method C.

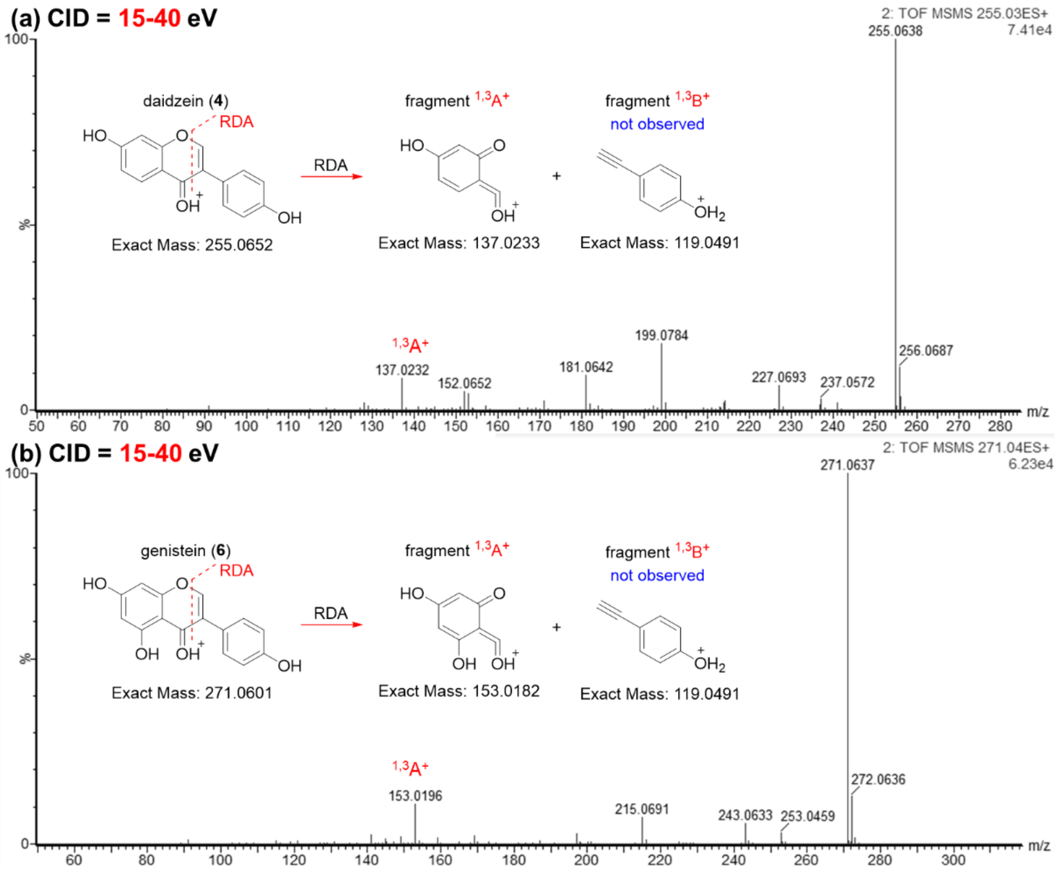

Notes: The fragments $^{1,3}A^{+}$ and $^{1,3}B^{+}$ yielded by retro-Diels–Alder (RDA) cleavage are key diagnostic ions of isoflavones, which can be used for structural inference of the following unknown isoflavone dimers. The remained ions are well explained in previous studies [14,15].

Figure S11. The MS–MS analysis of cattleyaisoflavone A (**1**). (a) Proposed fragmental pathway of **1**, and corresponding MS–MS spectra derived from (b) 15–40 eV and (c) 30–50 eV of CID. UHPLC–(+)HRMS–ESI analysis followed analytical method C.

Notes: The dimer **1** is linked by C–C bond between two A rings, and even high CID cannot destroy this stable bond to release monomer fragments. The direct RDA fragments were not observed, while ion *m*/*z* 375 corresponds with [**4** + ^{1,3}A − CO_2]$^+$. Besides, some ions resulting from methyl or methoxy group cleavage were observed. Overall, the fragments induced by 30–50 eV CID were limited, indicating the solid skeleton of this dimer.

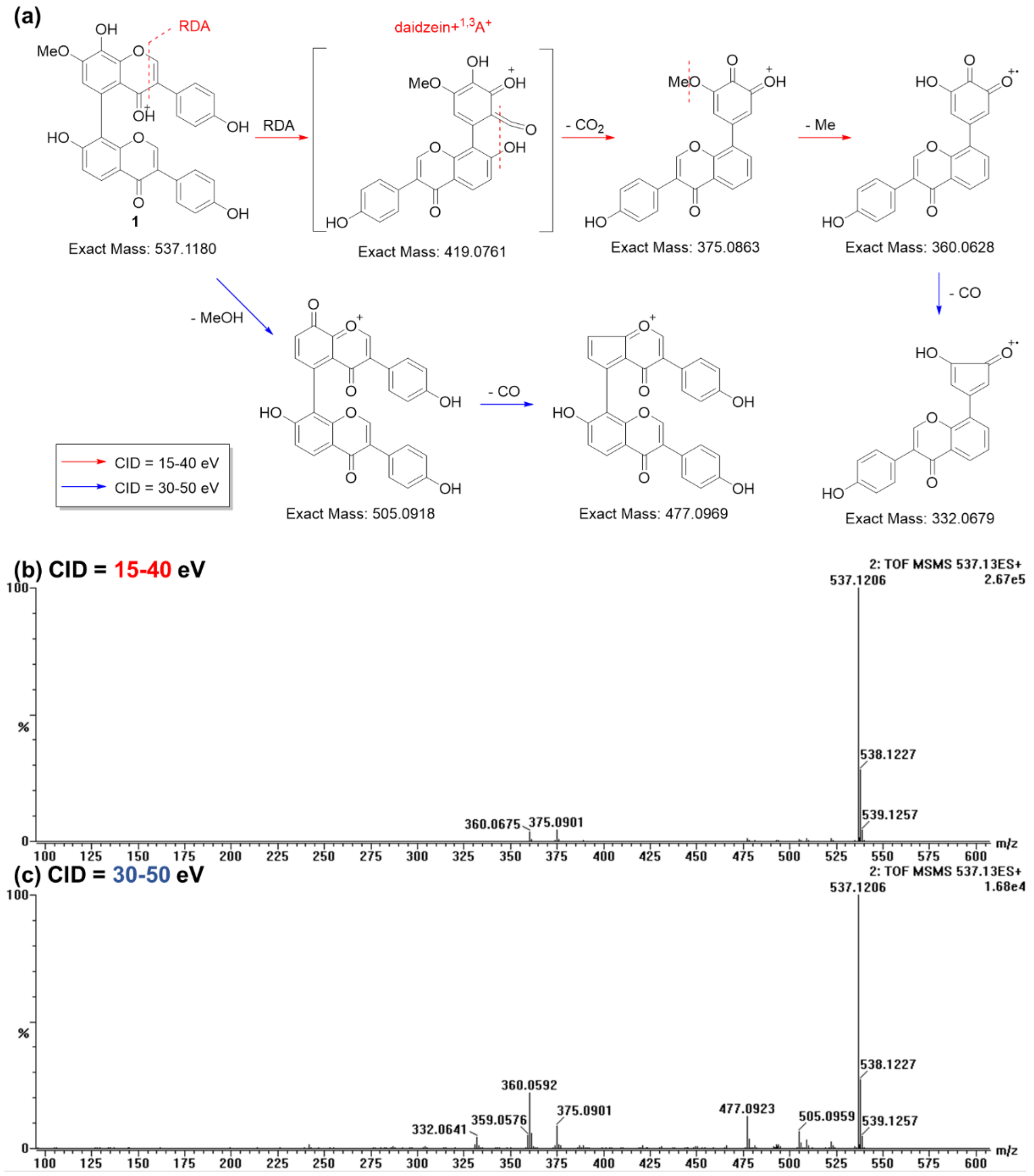

Figure S12. The MS–MS analysis of cattleyaisoflavone B (**2**). (a) Proposed fragmental pathway of **2**, and corresponding MS–MS spectra derived from (b) 15–40 eV and (c) 30–50 eV of CID. UHPLC–(+)HRMS–ESI analysis followed analytical method C.

Notes: The asymmetric dimer **2** was linked by C–C bond between ring A and ring B, allowing two different RDA cleavages (b_1 and b_2) and resulting in corresponding diagnostic ions ($[\mathbf{4} + {}^{1,3}B]^+$, $[\mathbf{4} + {}^{1,3}A]^+$, and $[{}^{1,3}A]^+$). Besides the classical RDA pathway of isoflavone, dimer **2** also displayed atypical $[\mathbf{4} + B]^+$ fragment from ring B cleavage, and $[\mathbf{4} + {}^{2,3}B]^+$ fragment from cleavage **c** (Figure S12a). These ions are helpful for structural determination of unknown isoflavone dimers.

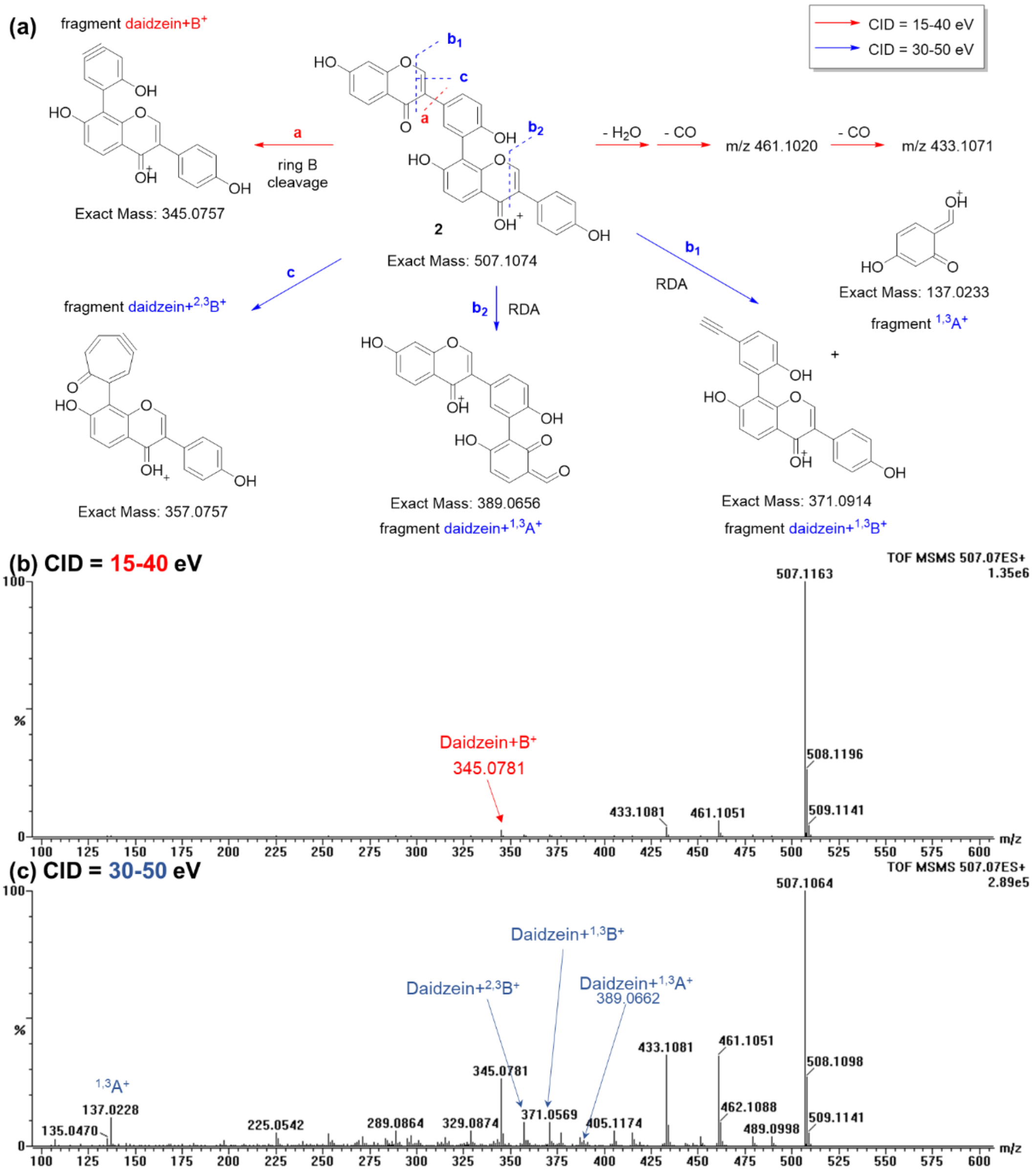

Figure S13. The MS–MS analysis of cattleyaisoflavone C (**3**). (a) Proposed fragmental pathway of **3**, and corresponding MS–MS spectra derived from (b) 15─40 eV and (c) 30─50 eV of CID. UHPLC–(+)HRMS–ESI analysis followed analytical method C.

Notes: The dimer **3** is linked by C-O bond of two daidzein (**4**) units, which is easy to cleave under CID 15–40 eV and form corresponding monomer ions. Because this C-O bond cleavage is more favorable than RDA cleavage, trace fragments were detectable in *m*/*z* region 255 to 507, showing significant difference with C–C-linked dimers (**1** and **2**). When increasing CID to 30–50 eV, the monomer ions *m*/*z* 253 displayed further fragmental pathways similar with **4**.

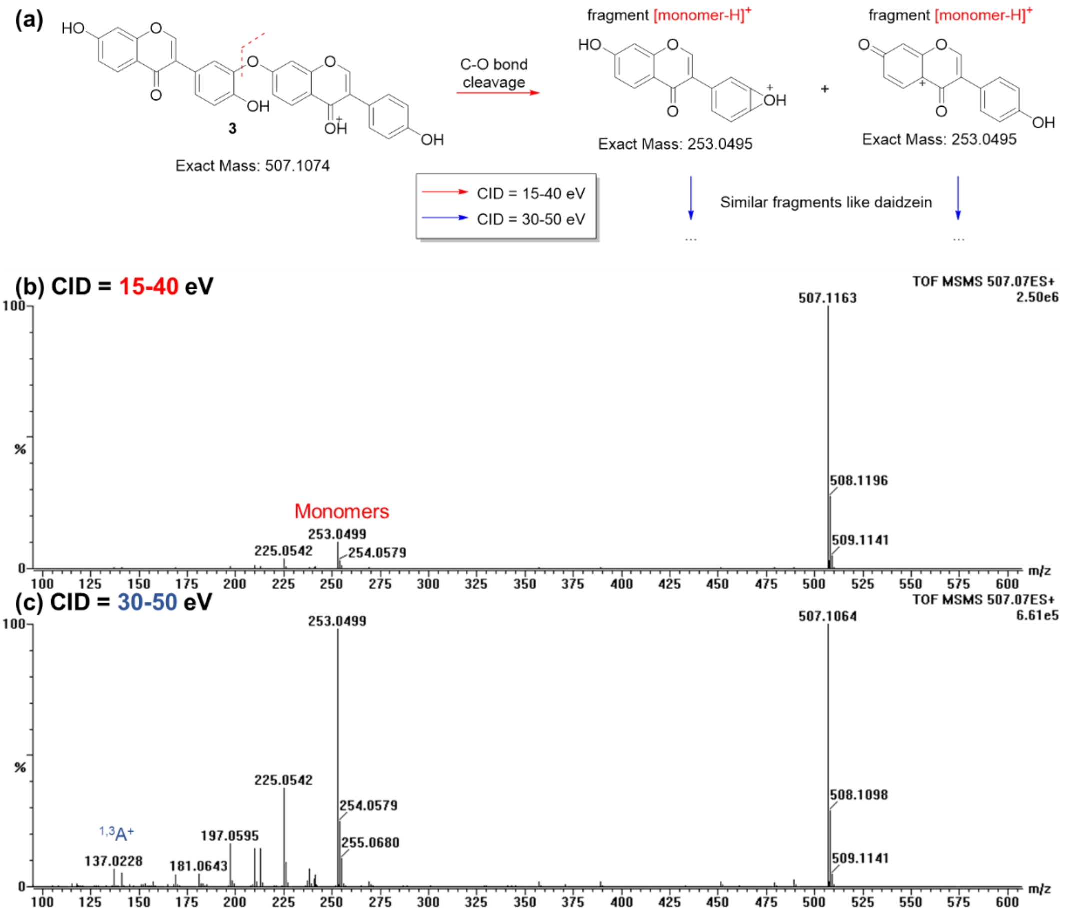

Figure S14. The MS–MS analysis of **5**. (a) Proposed fragmental pathway of **5**, and corresponding MS–MS spectra derived from (b) 15–40 eV and (c) 30–50 eV of CID. UHPLC–(+)HRMS–ESI analysis followed analytical method C.

The structural inference of 5: In contrast with **3**, the abundant fragments in *m*/*z* region 255 to 507 indicated that **5** is a dimer linked by a C–C bond. Though low CID energy (15–40 eV) was used, the characteristic ions assigned as [**4** + ^{1,3}B]$^+$, [**4** + ^{2,3}B]$^+$, and [^{1,3}A]$^+$ were observed in clear, which was different from dimer **2**. The absence of any [**4** + ^{1,3}A]$^+$ ions suggested that this C-C linkage should be located on two B rings. In consideration of the radical mechanism, **5** was assigned as a 3',3'-linked dimer. This symmetric skeleton also matched the high abundance of RDA-derived ions under low CID energy.

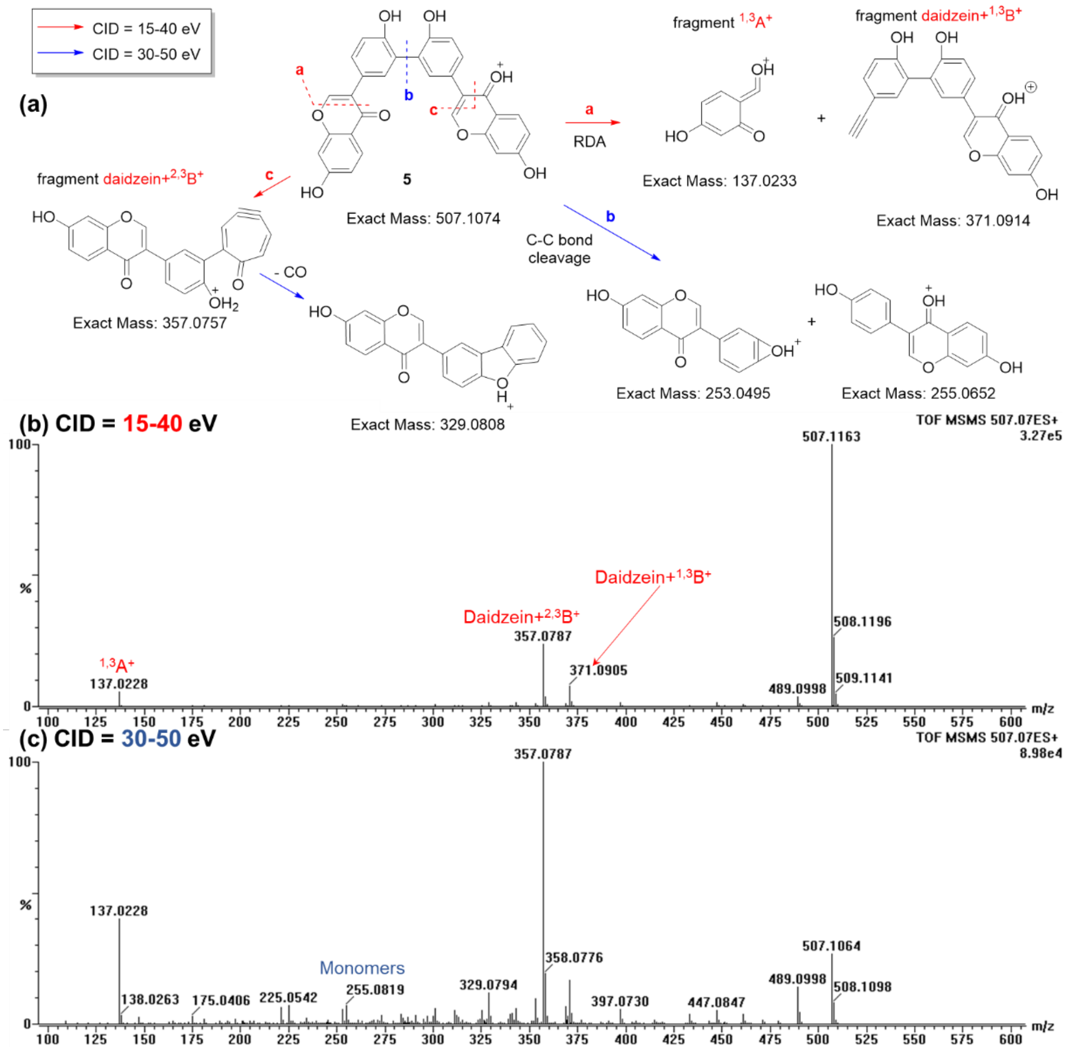

Figure S15. The MS–MS analysis of dimers derived from genistein (**6**). (a) Four peaks for dimers **d1-4** were observed from bioassay sample using substrate **6** and CYP158C1 (analytical method D). (b–e) MS–MS spectra of **d1-4** resulted in (f) proposed structures and (g–i) corresponding fragmental pathways.

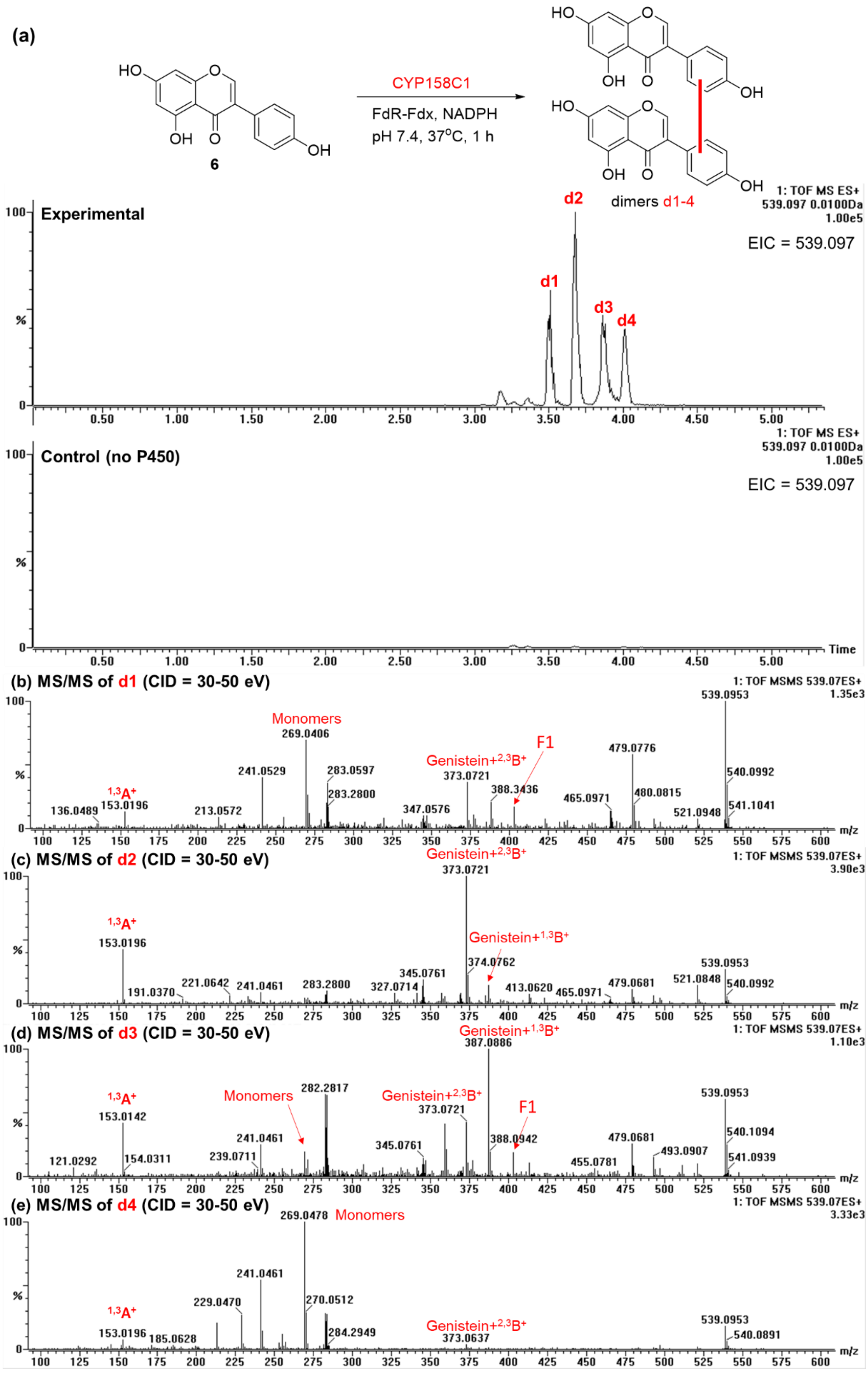

Notes: Four peaks were assigned to dimers **d1-4** derived from **6** based on their MS–MS spectra. The dimer **d4** was suggested to be a C–O-linked product by the comparison with **3**, while the coupling sites were unknown. And **d2** was likely to be a 3’–3’-linked symmetric dimer, because of its preference for fragments [**6** + $^{1,3}B]^+$, [**6** + $^{2,3}B]^+$, and $[^{1,3}A]^+$, which is similar with compound **5**. The dimers **d1** and **d3** might be linked between rings A and B, due to the observed [**6** + $^{1,3}B]^+$ and [**6** + $^{1,3}A]^+$ related ions.

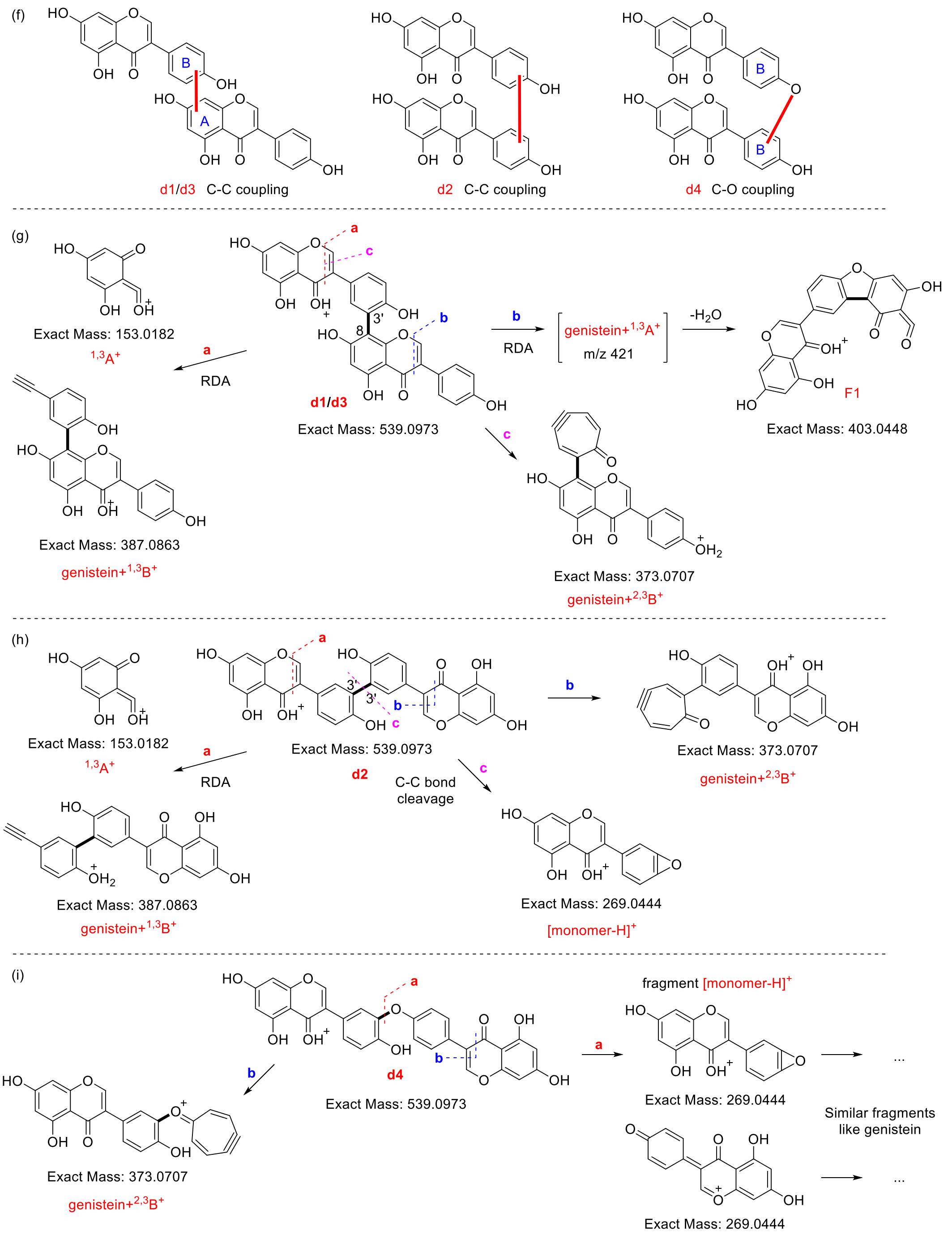

Figure S16. The MS–MS analysis of dimers derived from flavone **10**. (a) Three peaks for dimers **d5-7** were observed from bioassay sample using substrate **9** and CYP158C1 (analytical method D). (b–d) MS–MS spectra of **d5-7** resulted in (e) proposed structures and (f–h) corresponding fragmental pathways.

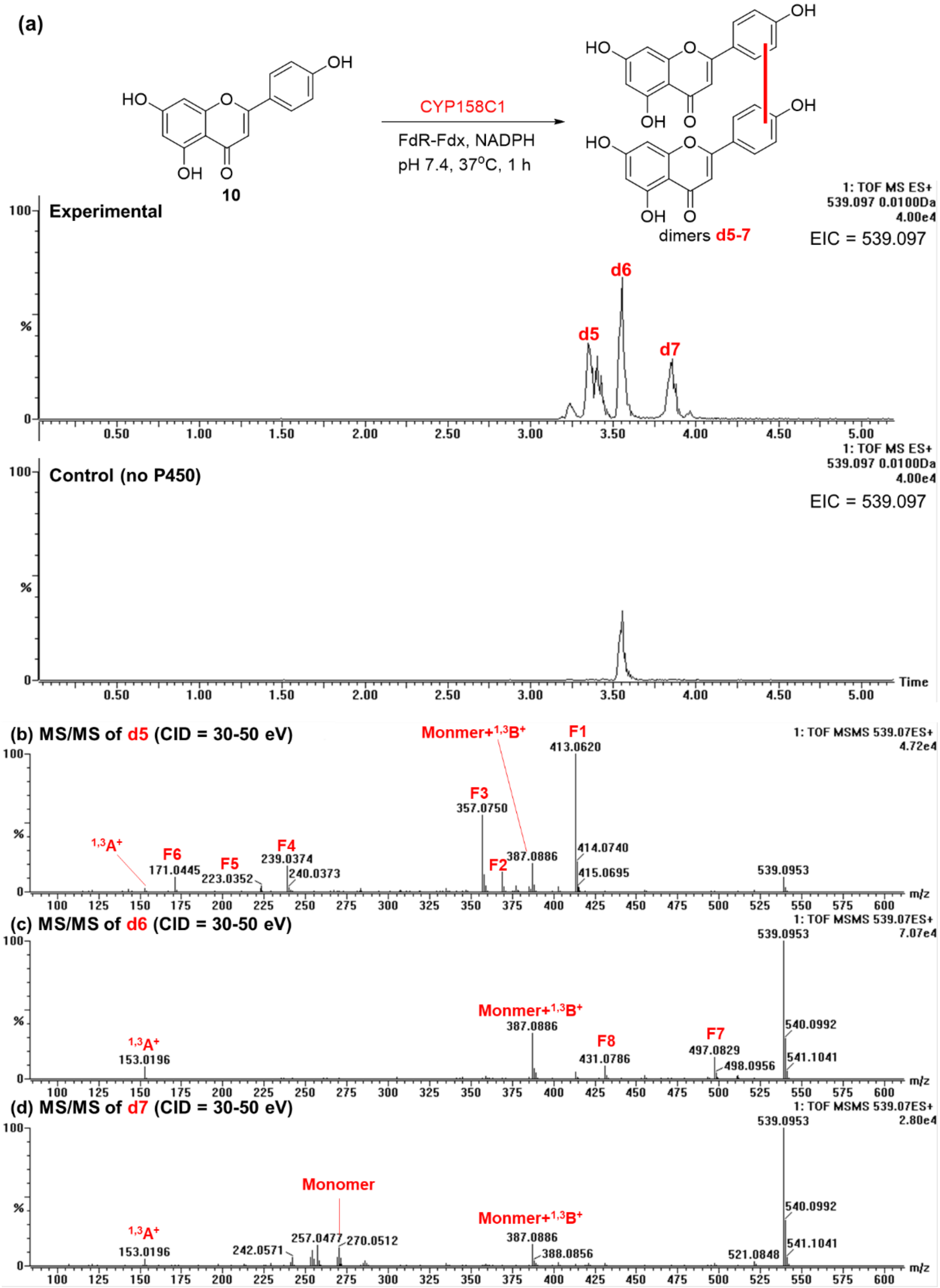

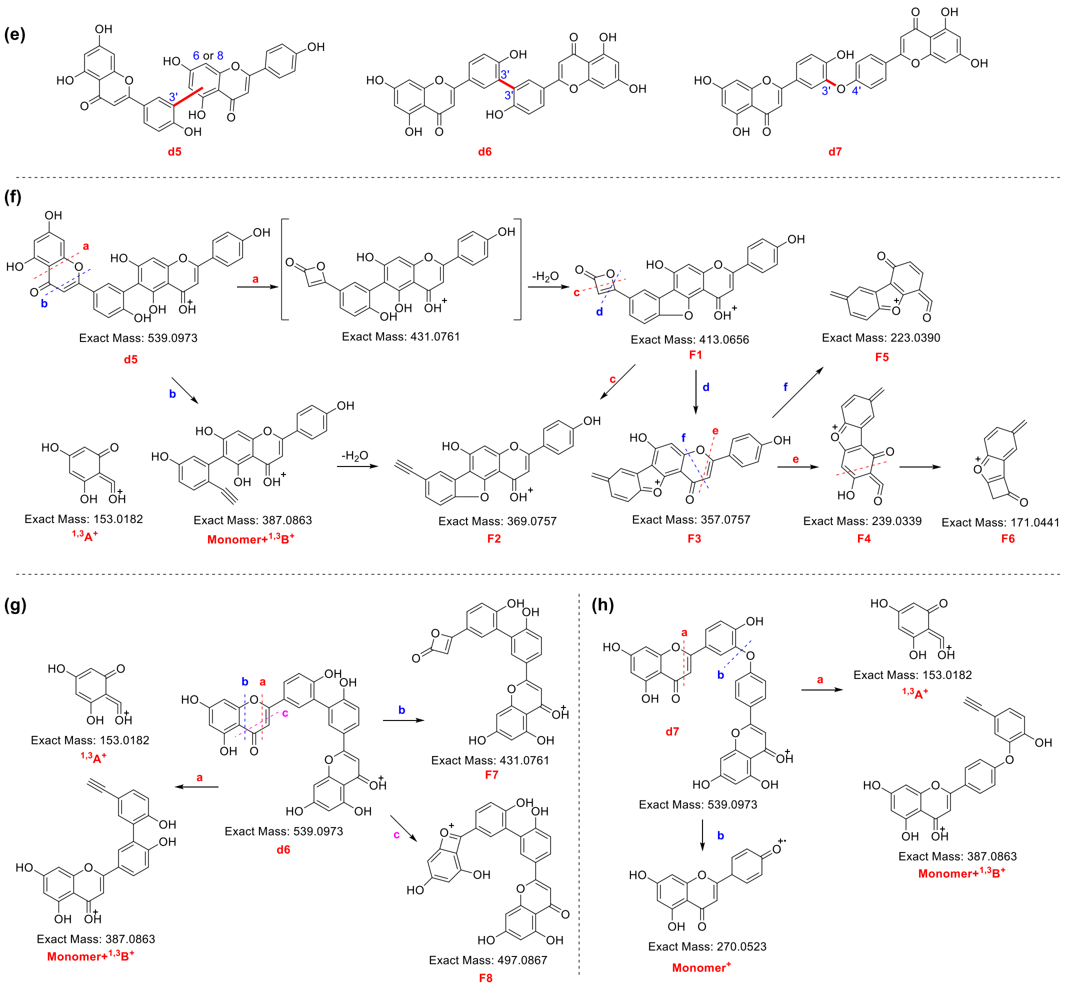
(e)
d5
d6
d7
(f)
Exact Mass: 539.0973
d5
Exact Mass: 431.0761
-H2O
Exact Mass: 413.0656
F1
Exact Mass: 223.0390
F5
Exact Mass: 153.0182
1,3A+
Exact Mass: 387.0863
Monomer+1,3B+
Exact Mass: 369.0757
F2
Exact Mass: 357.0757
F3
Exact Mass: 239.0339
F4
Exact Mass: 171.0441
F6
(g)
Exact Mass: 153.0182
1,3A+
Exact Mass: 539.0973
d6
Exact Mass: 431.0761
F7
Exact Mass: 387.0863
Monomer+1,3B+
Exact Mass: 497.0867
F8
(h)
d7
Exact Mass: 539.0973
Exact Mass: 270.0523
Monomer+
Exact Mass: 153.0182
1,3A+
Exact Mass: 387.0863
Monomer+1,3B+

Figure S17. The MS–MS analysis of dimers derived from flavone **11**. (a) Three peaks for dimers **d8-10** were observed from bioassay sample using substrate **11** and CYP158C1 (analytical method D). (b–d) MS–MS spectra of **d8-10** resulted in (e) proposed structures and (f–h) corresponding fragmental pathways.

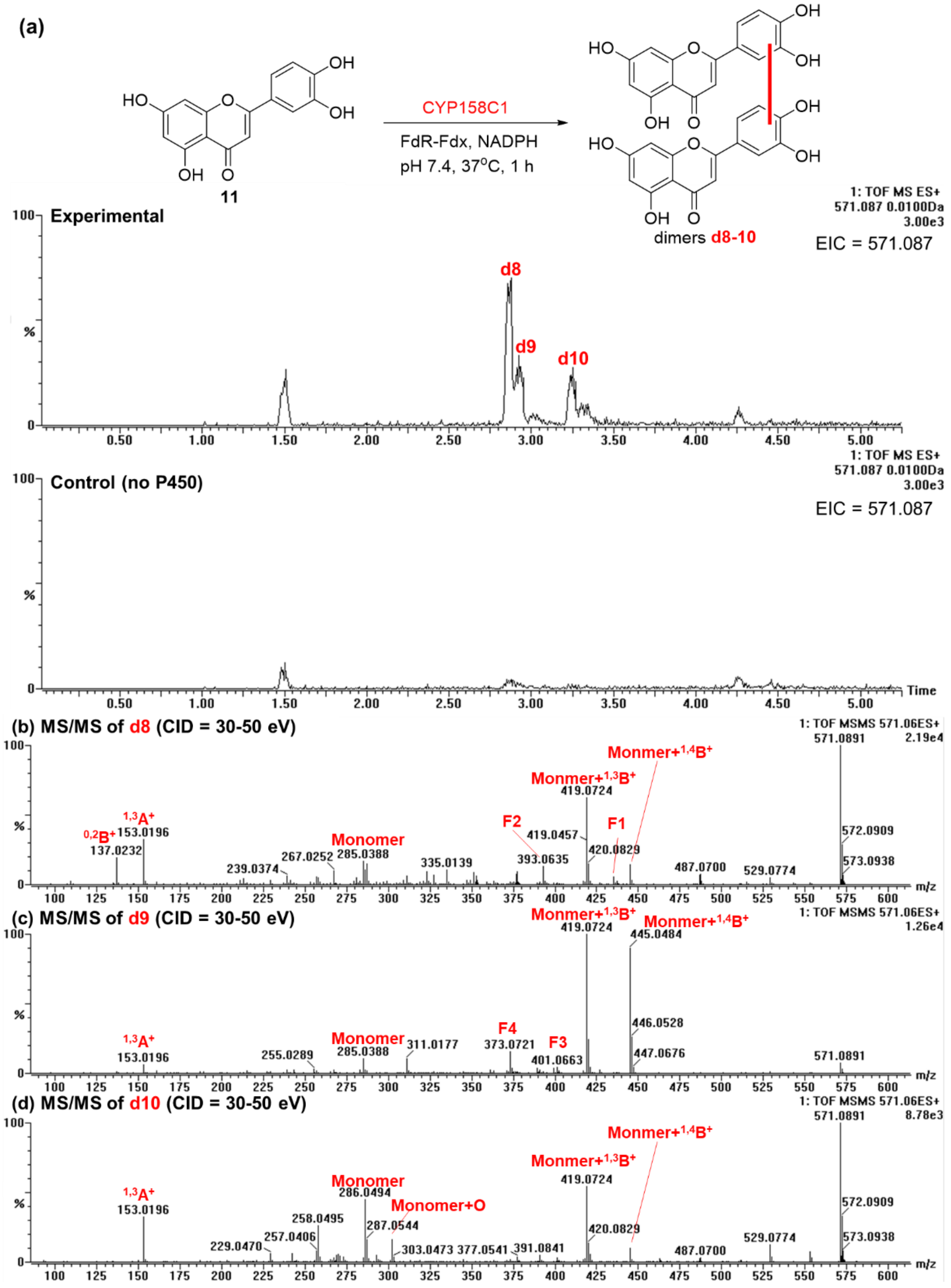

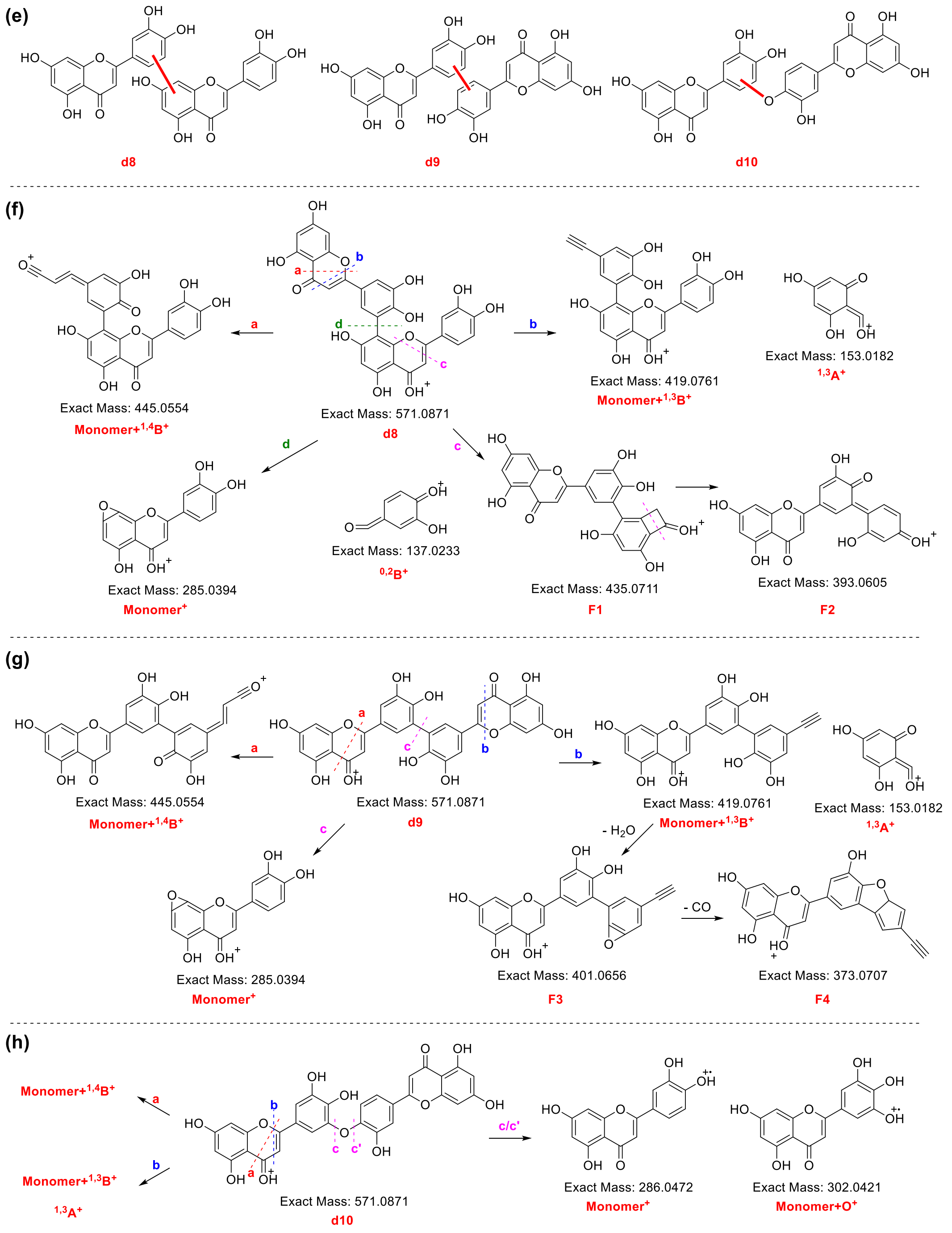
(e)
d8
d9
d10
(f)
Exact Mass: 445.0554
Monomer+^{1,4}B^{+}
Exact Mass: 571.0871
d8
Exact Mass: 419.0761
Monomer+^{1,3}B^{+}
Exact Mass: 153.0182
^{1,3}A^{+}
Exact Mass: 285.0394
Monomer^{+}
Exact Mass: 137.0233
^{0,2}B^{+}
Exact Mass: 435.0711
F1
Exact Mass: 393.0605
F2
(g)
Exact Mass: 445.0554
Monomer+^{1,4}B^{+}
Exact Mass: 571.0871
d9
Exact Mass: 419.0761
Monomer+^{1,3}B^{+}
Exact Mass: 153.0182
^{1,3}A^{+}
- H_2O
Exact Mass: 285.0394
Monomer^{+}
Exact Mass: 401.0656
F3
- CO
Exact Mass: 373.0707
F4
(h)
Monomer+^{1,4}B^{+}
Monomer+^{1,3}B^{+}
^{1,3}A^{+}
Exact Mass: 571.0871
d10
c/c'
Exact Mass: 286.0472
Monomer^{+}
Exact Mass: 302.0421
Monomer+O^{+}

Figure S18. The MS–MS analysis of dimer derived from flavonol **12**. (a) One peak for dimer **d11** was observed from bioassay sample using substrate **12** and CYP158C1 (analytical method D). (b) MS–MS spectrum and (c) proposed MS–MS pathway of one possible structure of **d11**.

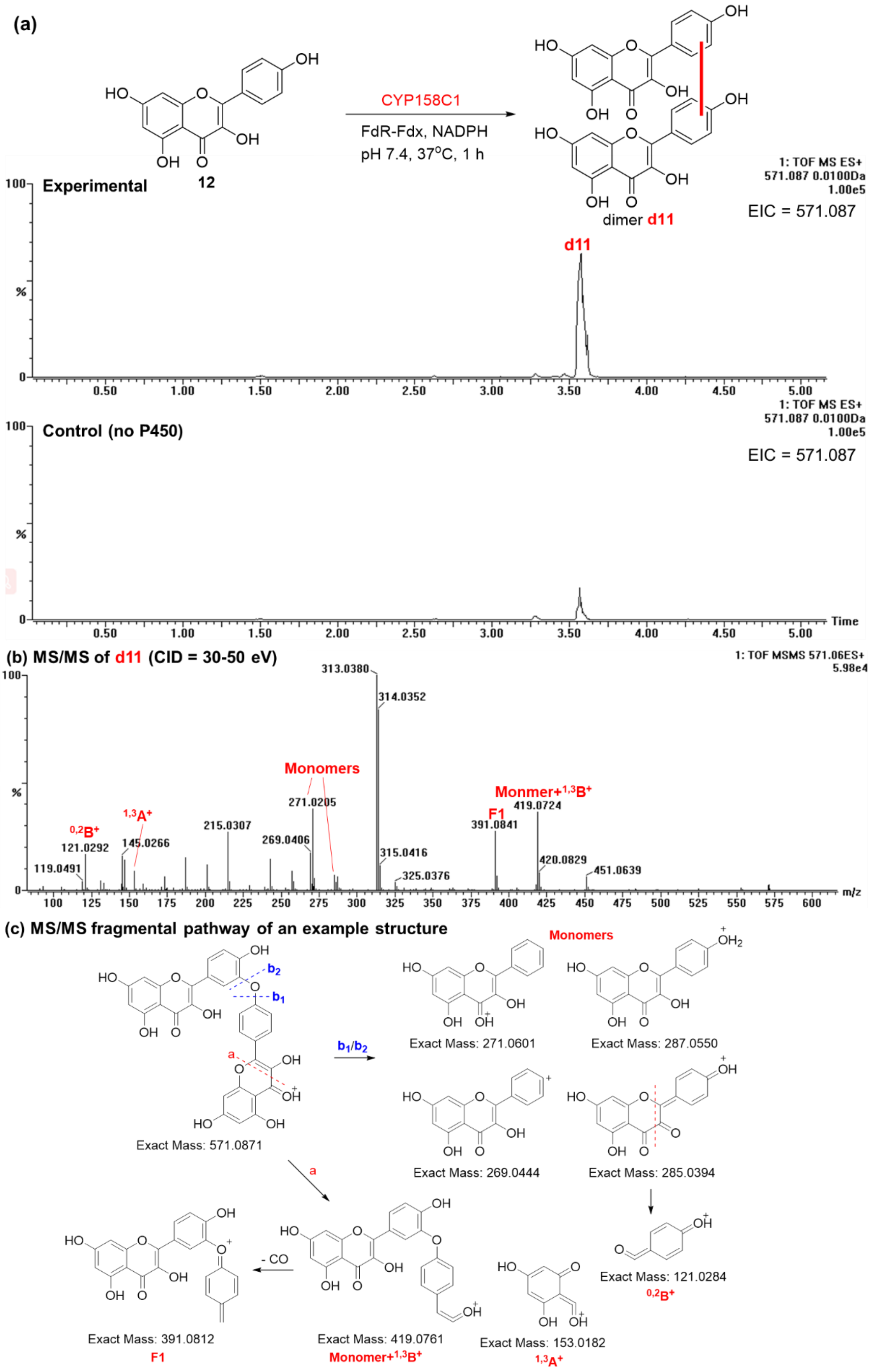

Figure S19. The MS–MS analysis of dimers derived from coumarin **18**. (a) Two peaks for dimers **d12-13** were observed from bioassay sample using substrate **18** and CYP158C1 (analytical method D). (b and c) MS–MS spectra of **d12-13** were used to identify their structures tentatively. (d) Proposed MS–MS pathway of one possible structure of **d12-13**.

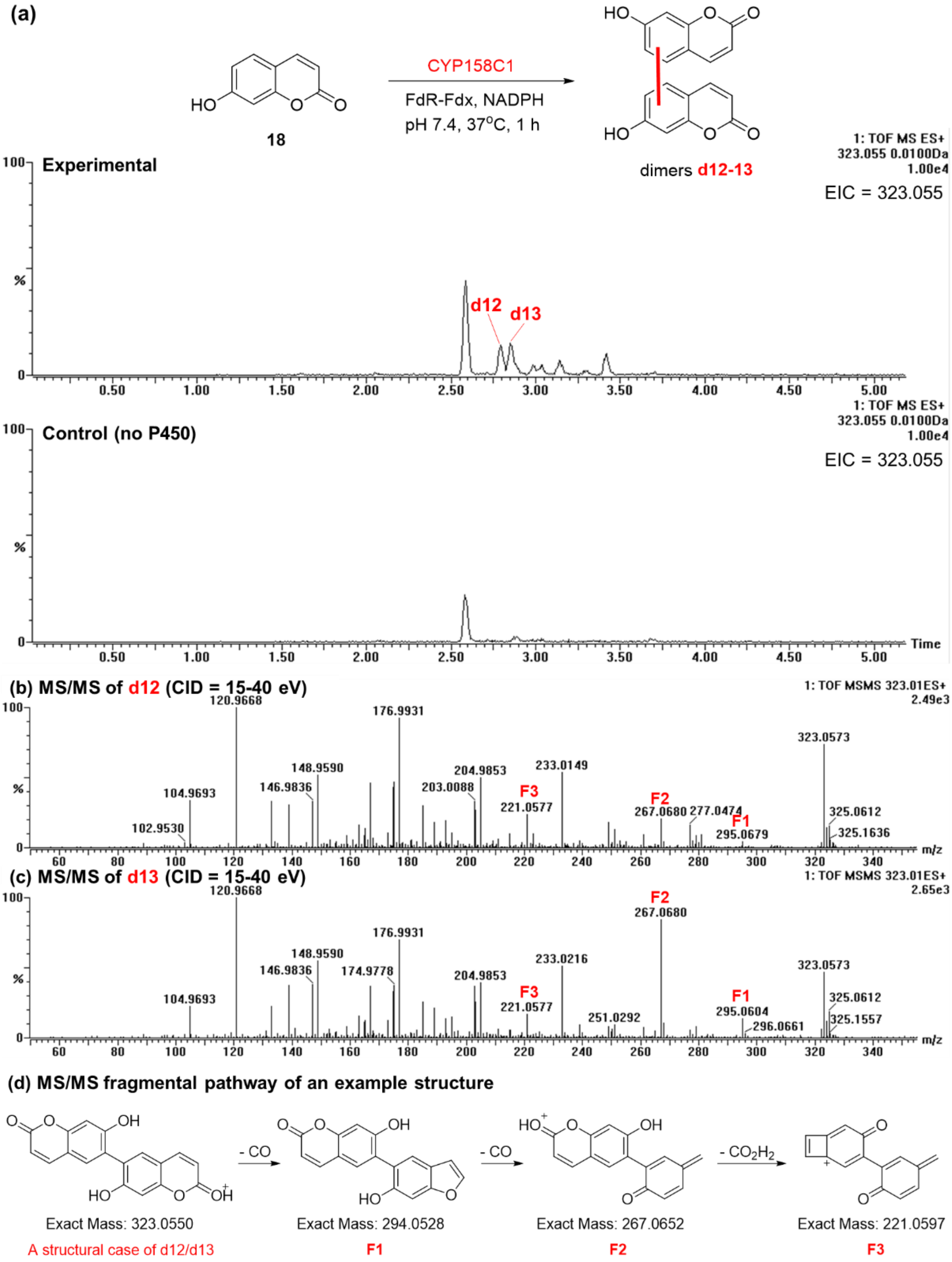

Figure S20. The MS–MS analysis of dimer derived from RVT. (a) One peak for dimer **d14** was observed from bioassay sample using substrate RVT and CYP158C1 (analytical method D). (b) MS–MS spectrum and (c) proposed MS–MS pathway of one possible structure of **d14**.

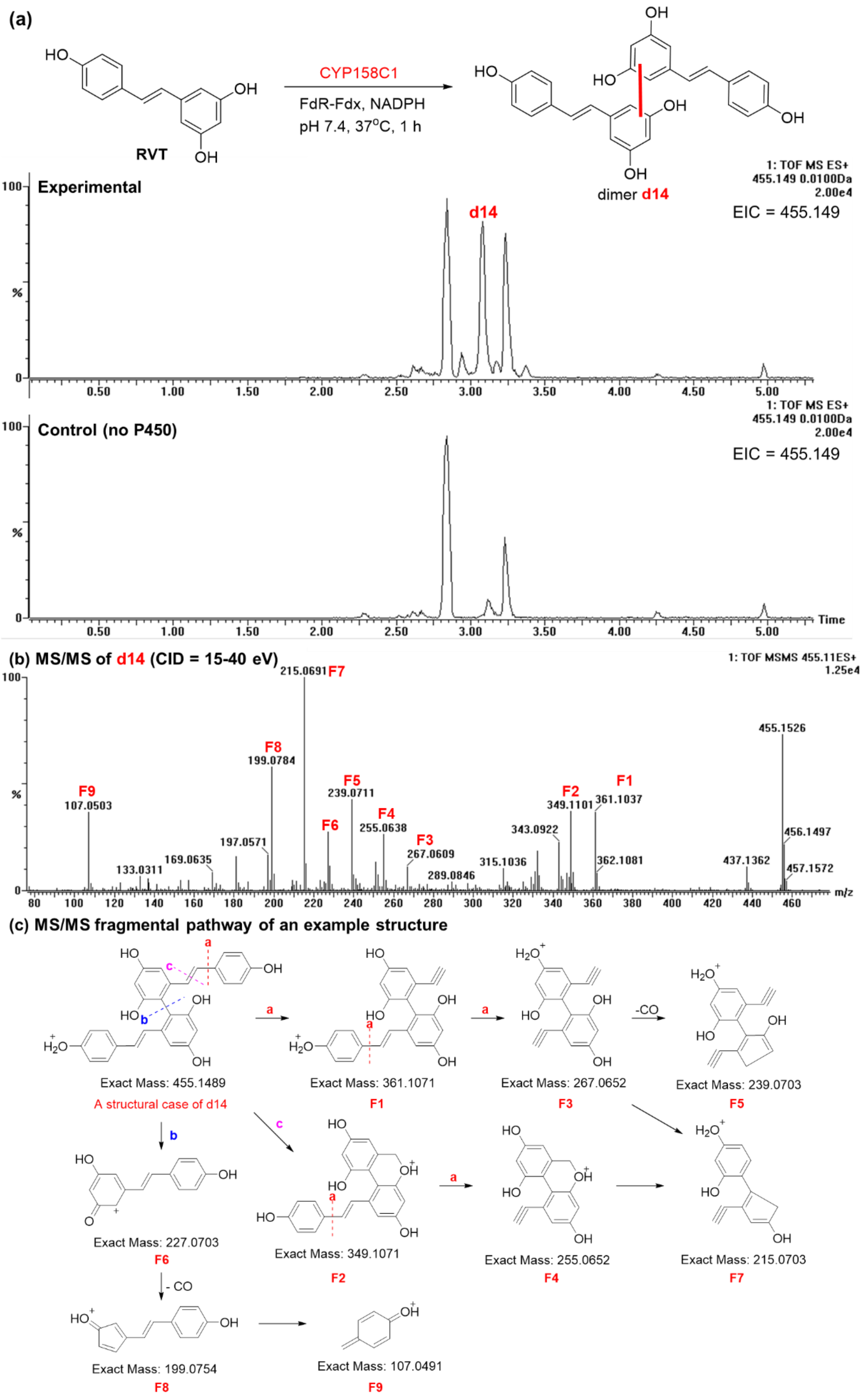

4. Supplementary figures part III: NMR data of compounds 1–3

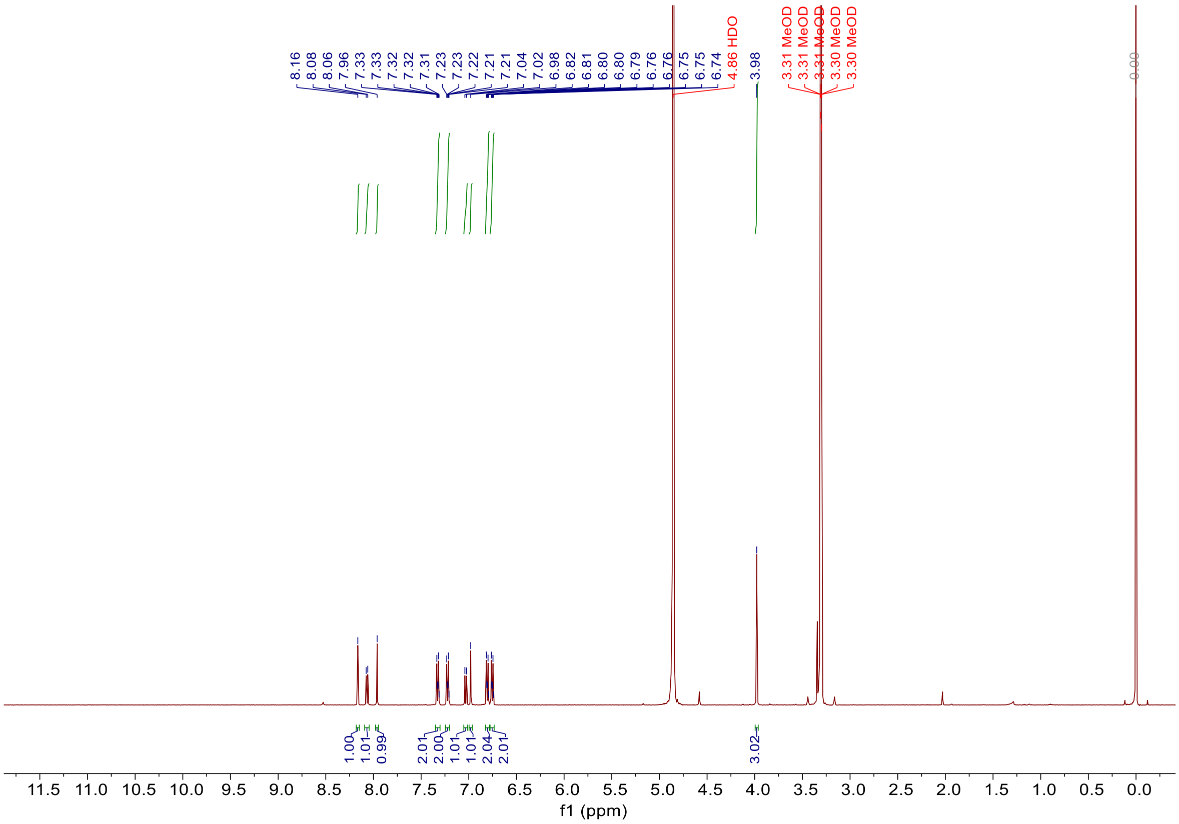

Figure S21. ^{1}H NMR spectrum (500 MHz, MeOH-d_4) of **1**.

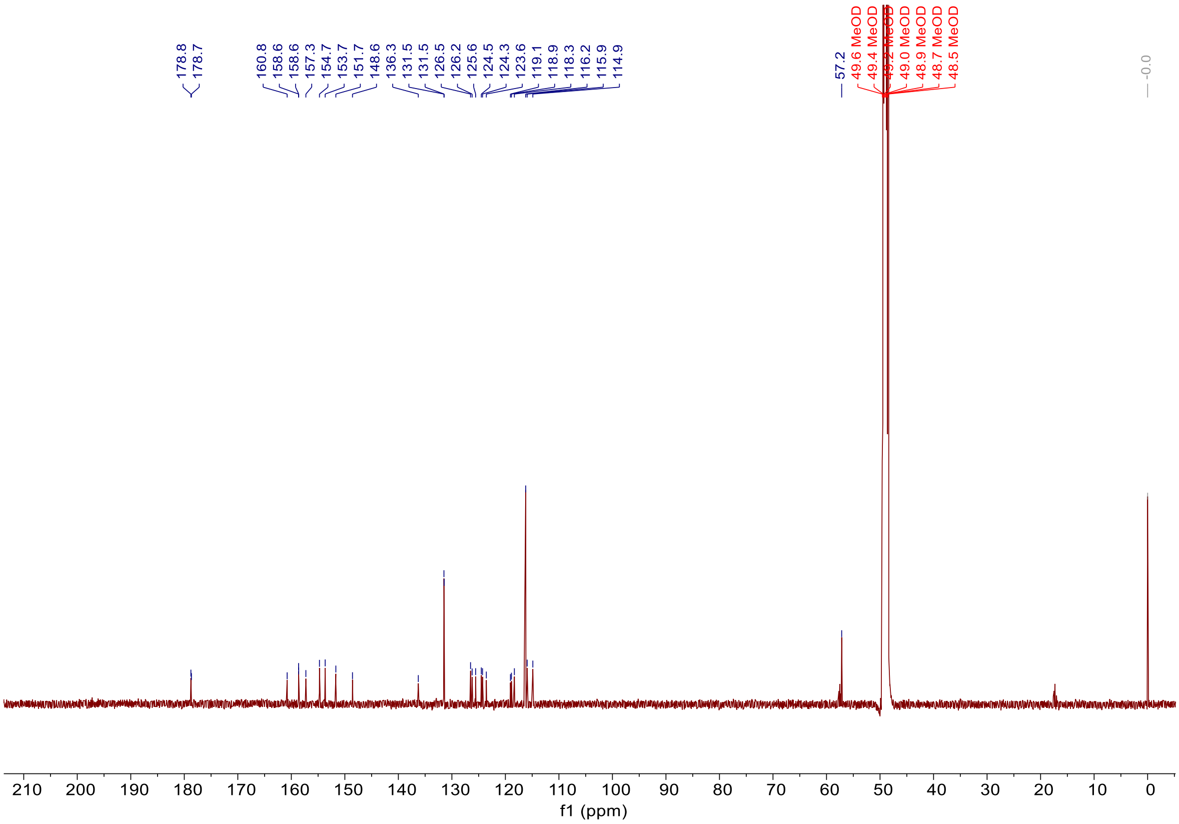

Figure S22. ^{13}C NMR spectrum (125 MHz, MeOH-d_4) of **1**.

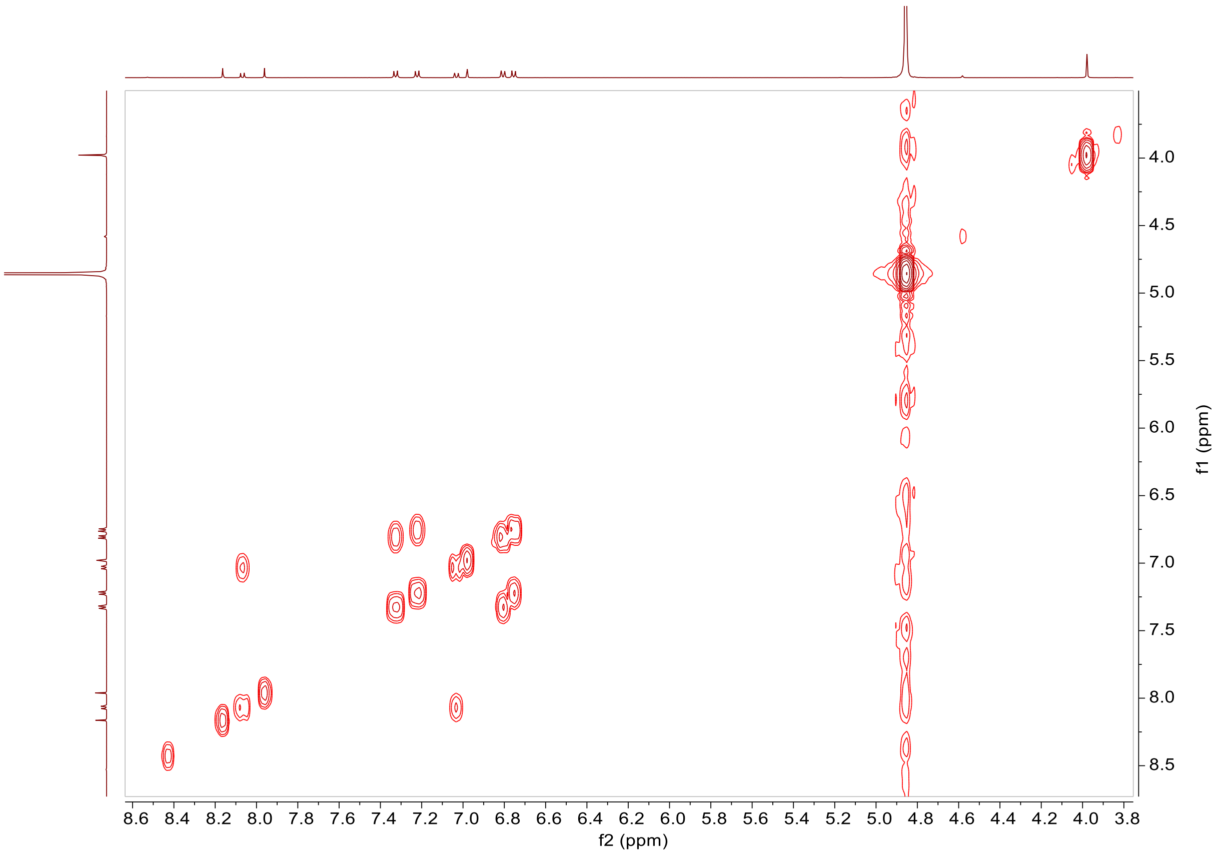

Figure S23. $^{1}H,^{1}H$-COSY spectrum (500 MHz, MeOH-*d*4) of **1**.

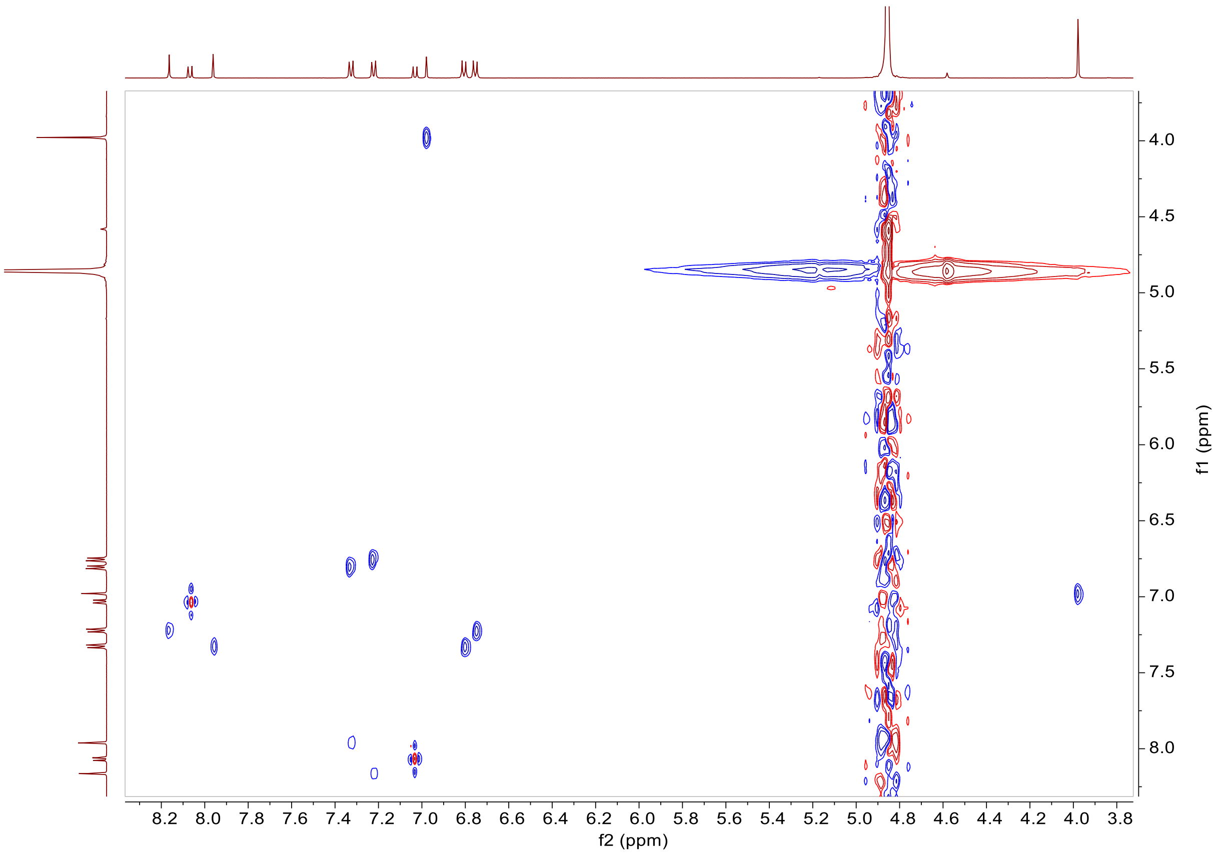

Figure S24. $^{1}H,^{1}H$-NOESY spectrum (500 MHz, MeOH-*d*4) of **1**.

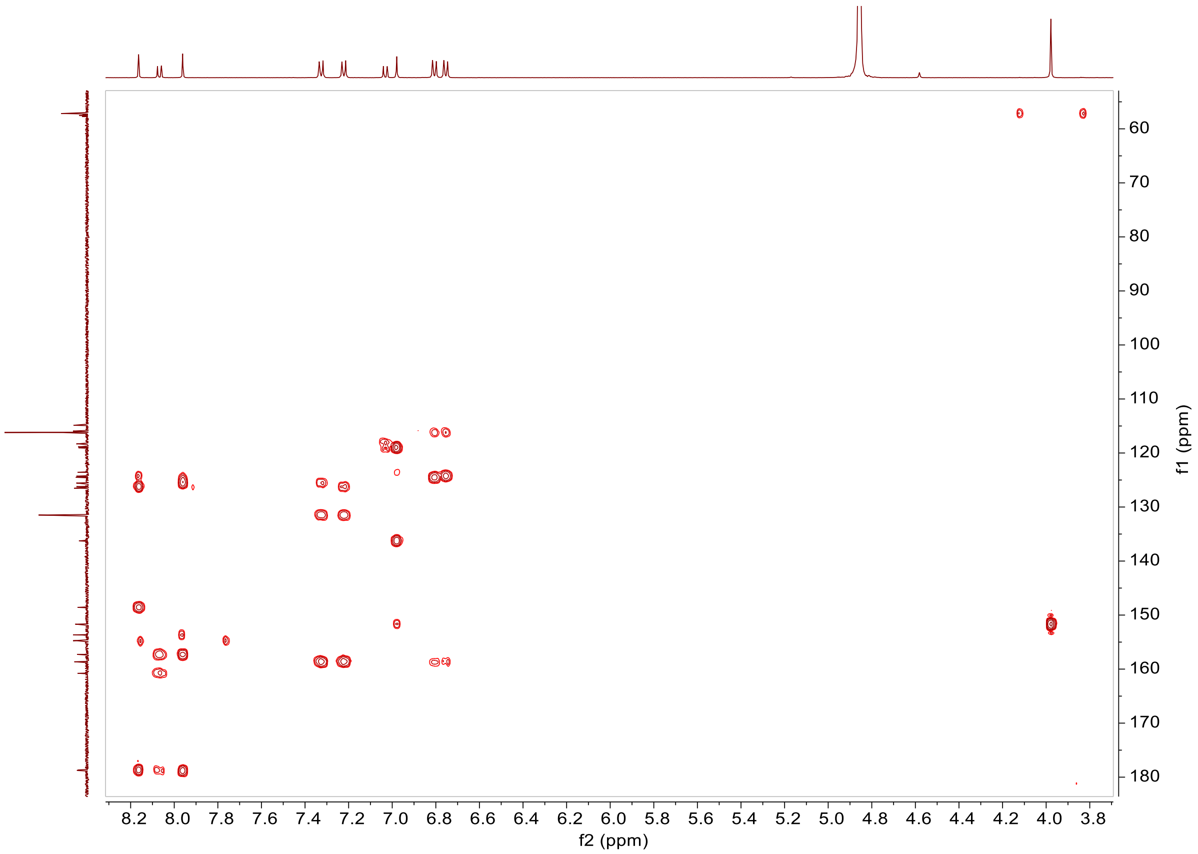

Figure S25. HMBC spectrum (500 MHz, MeOH-d_4) of **1**.

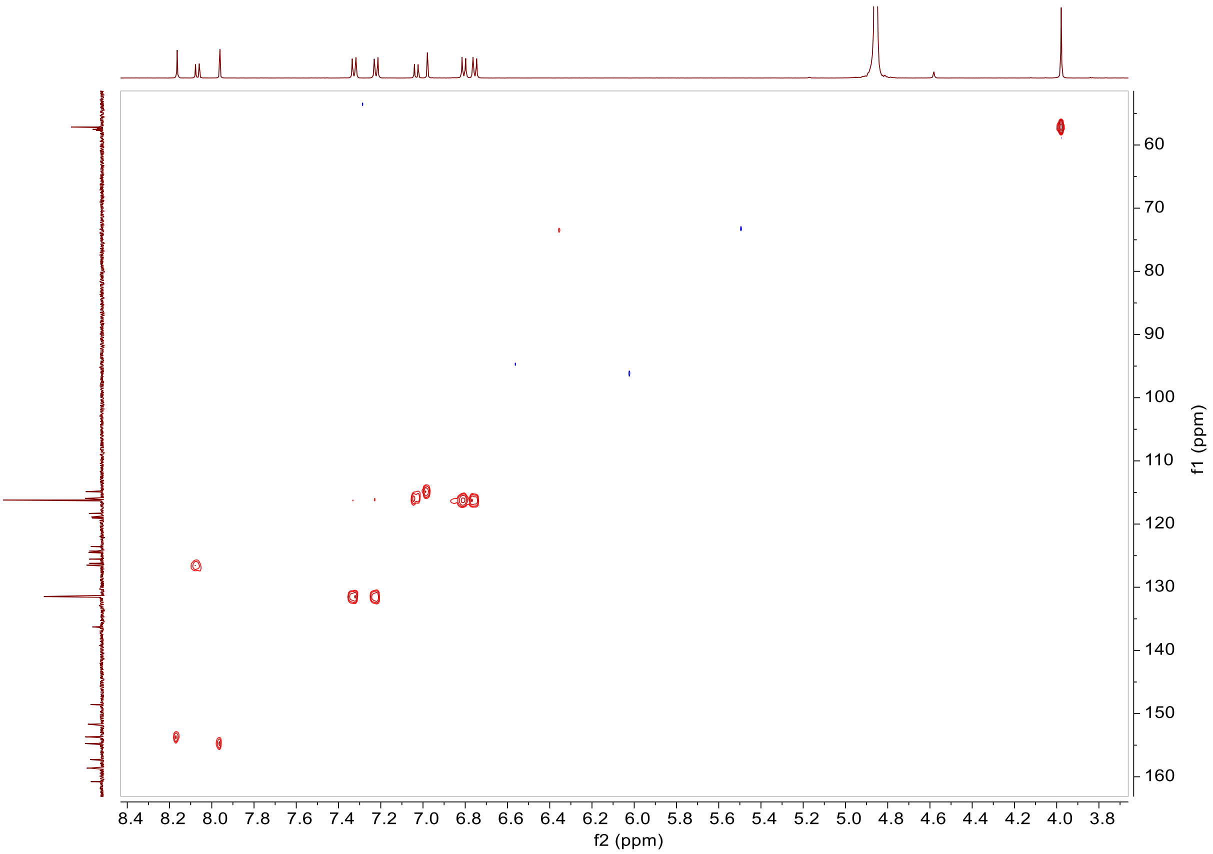

Figure S26. HSQC spectrum (500 MHz, MeOH-d_4) of **1**.

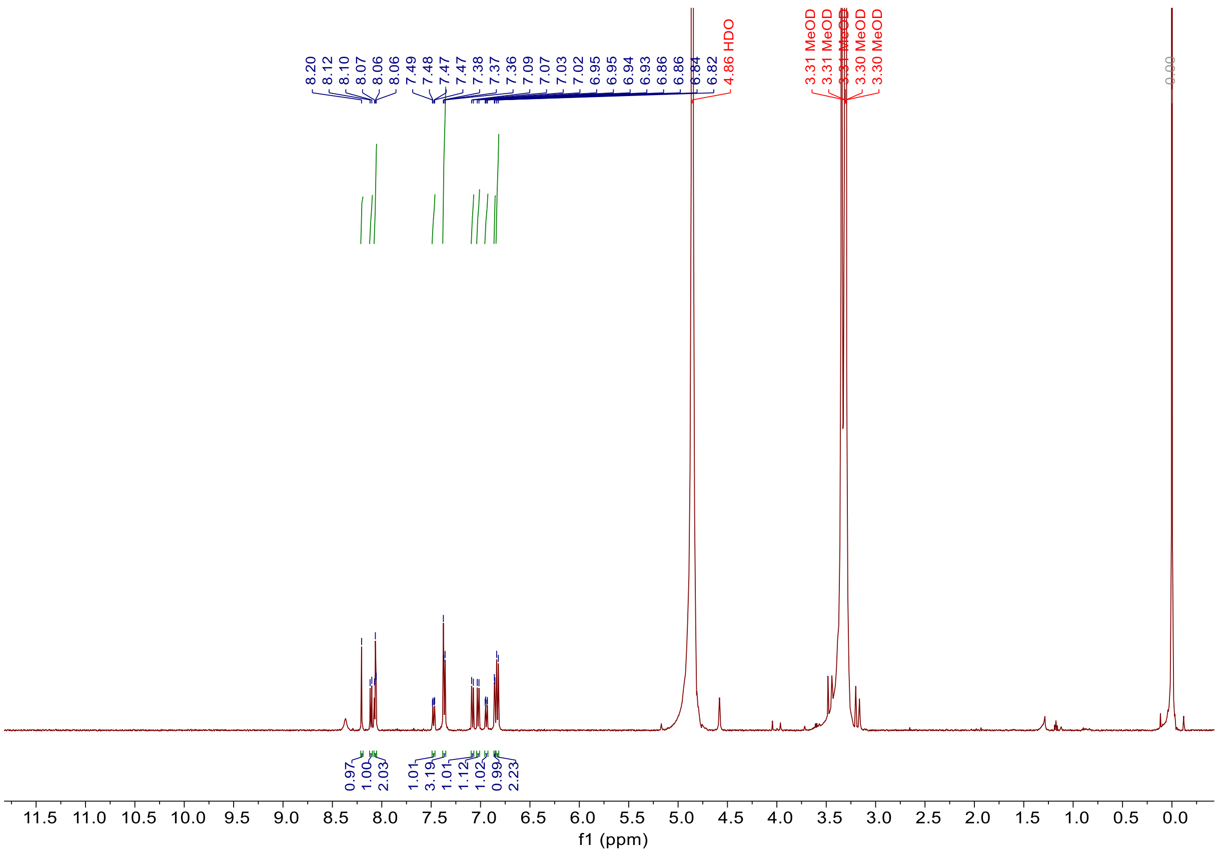

Figure S27. ^{1}H NMR spectrum (500 MHz, MeOH-d_4) of **2**.

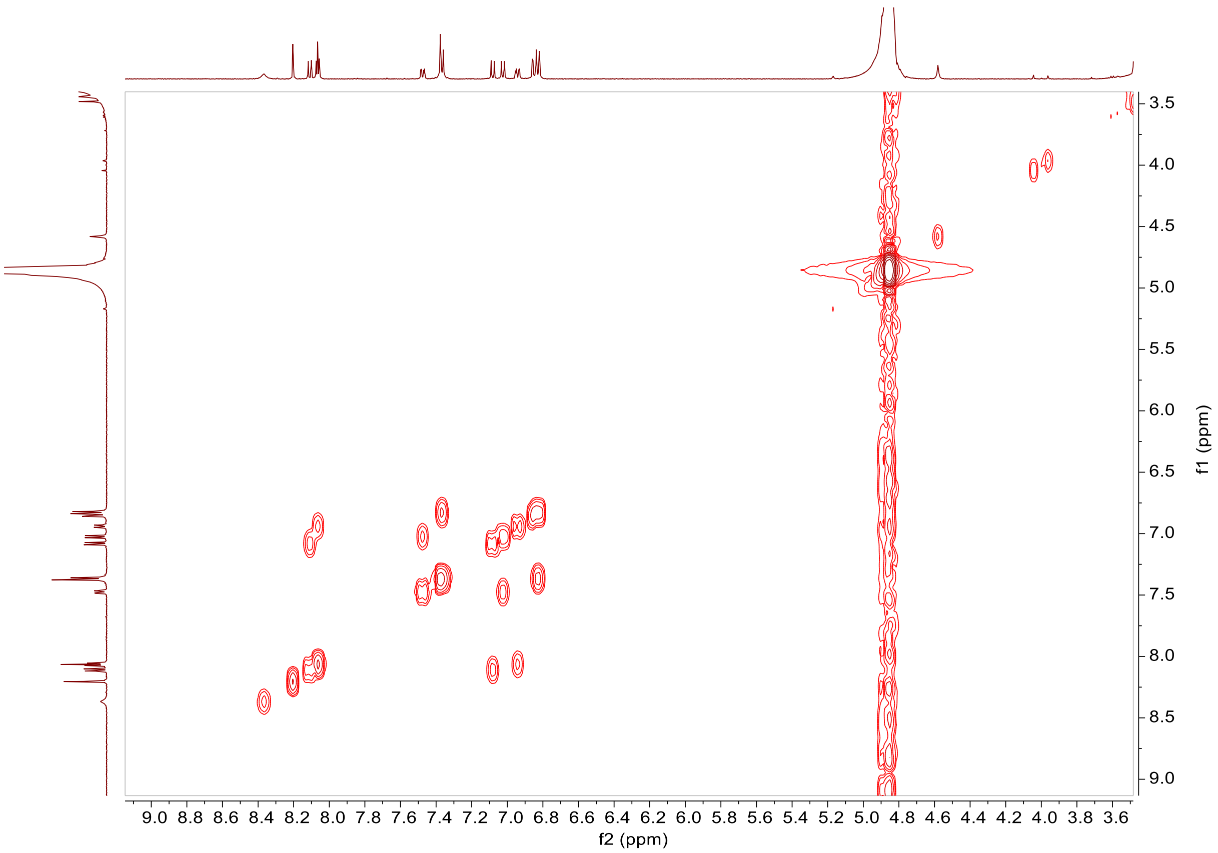

Figure S28. ^{1}H,^{1}H-COSY spectrum (500 MHz, MeOH-d_4) of **2**.

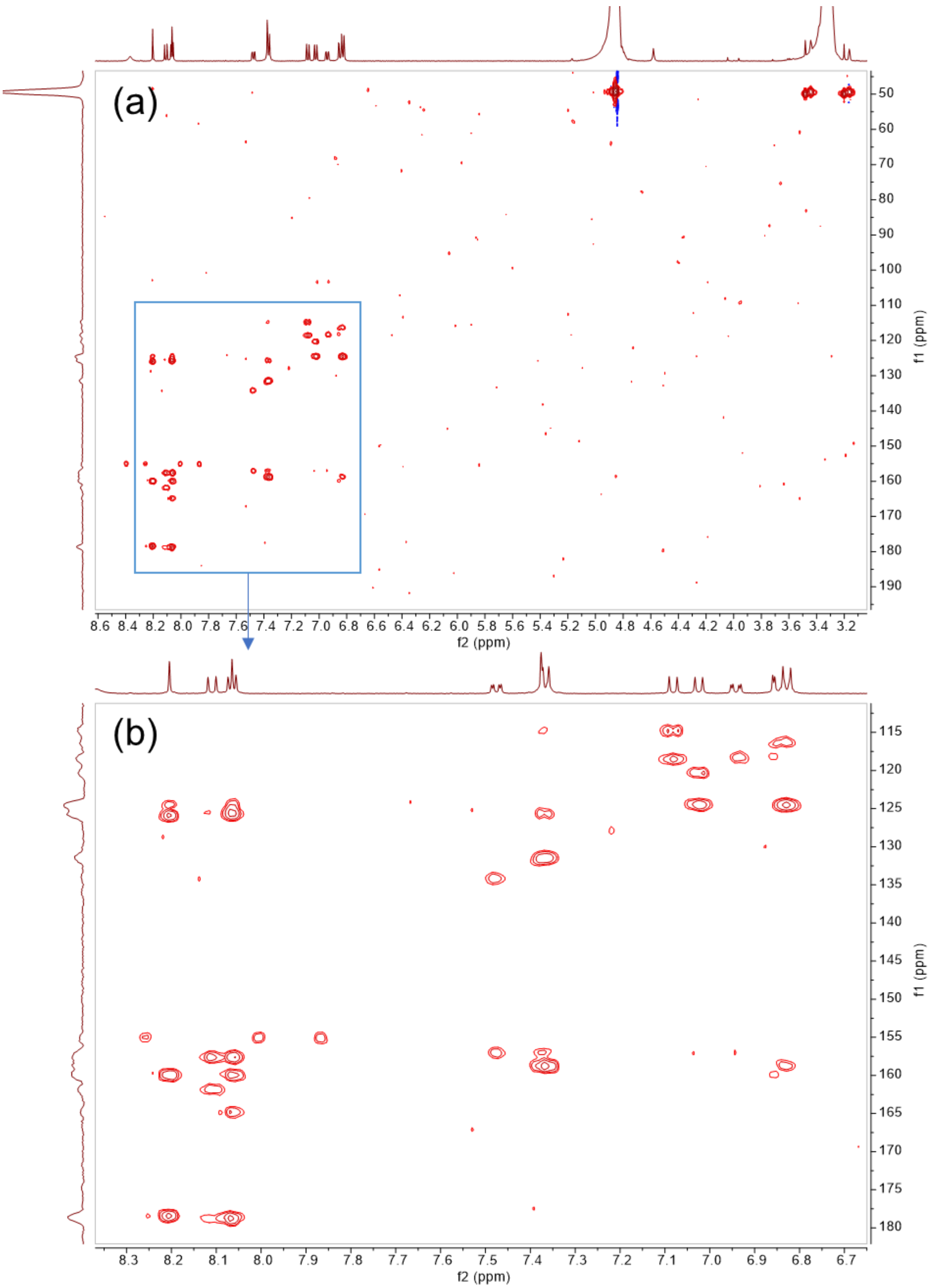

Figure S29. (a) HMBC spectrum (500 MHz, MeOH-d_4) of **2**; (b) Amplificated region in blue box.

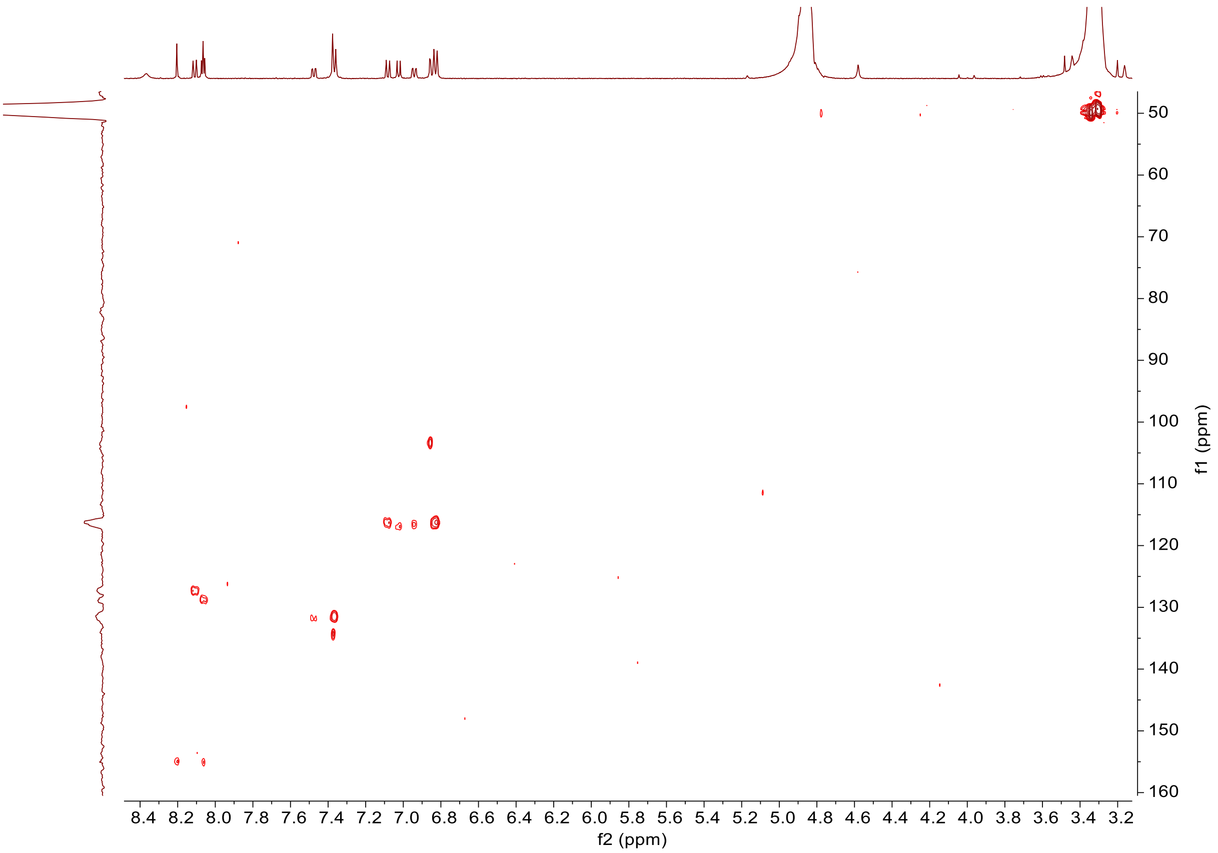

Figure S30. HSQC spectrum (500 MHz, MeOH-d_4) of **2**.

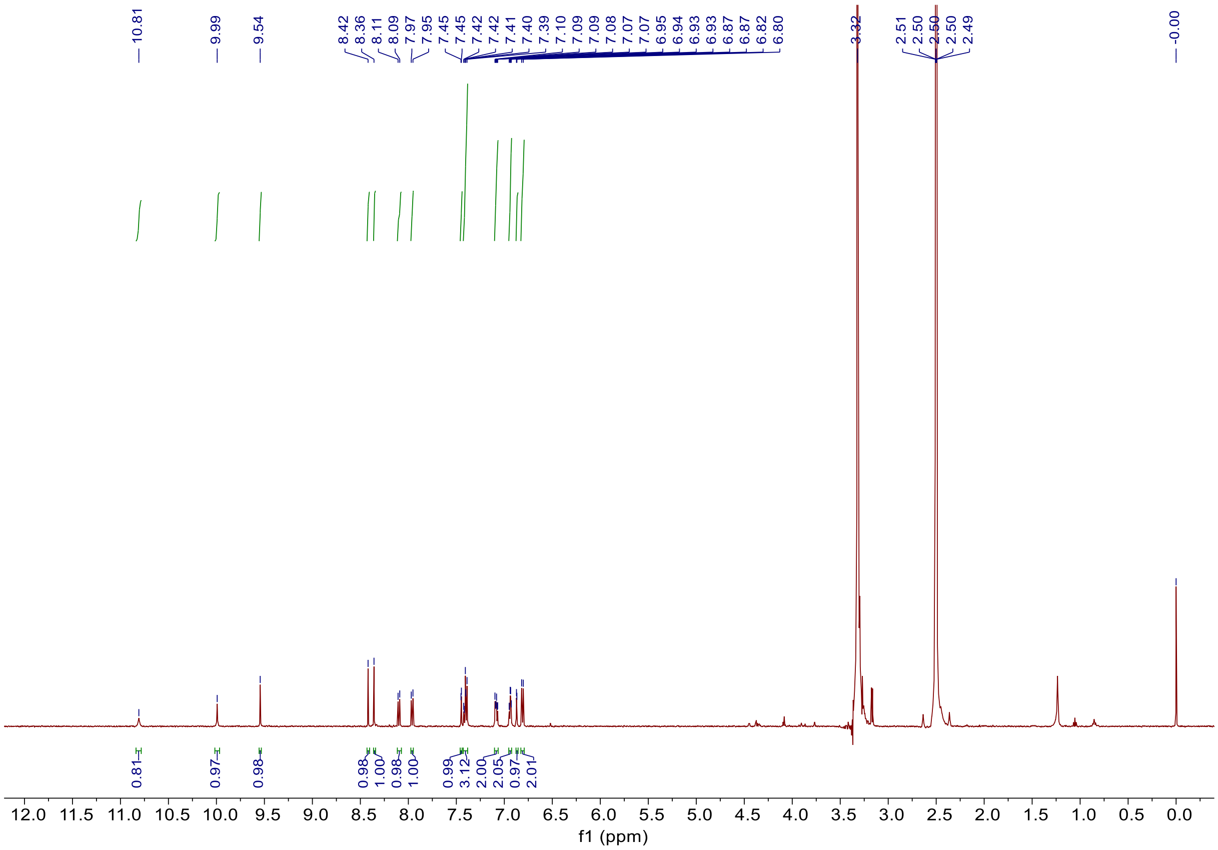

Figure S31. ^{1}H NMR spectrum (500 MHz, DMSO-d_6) of **3**.

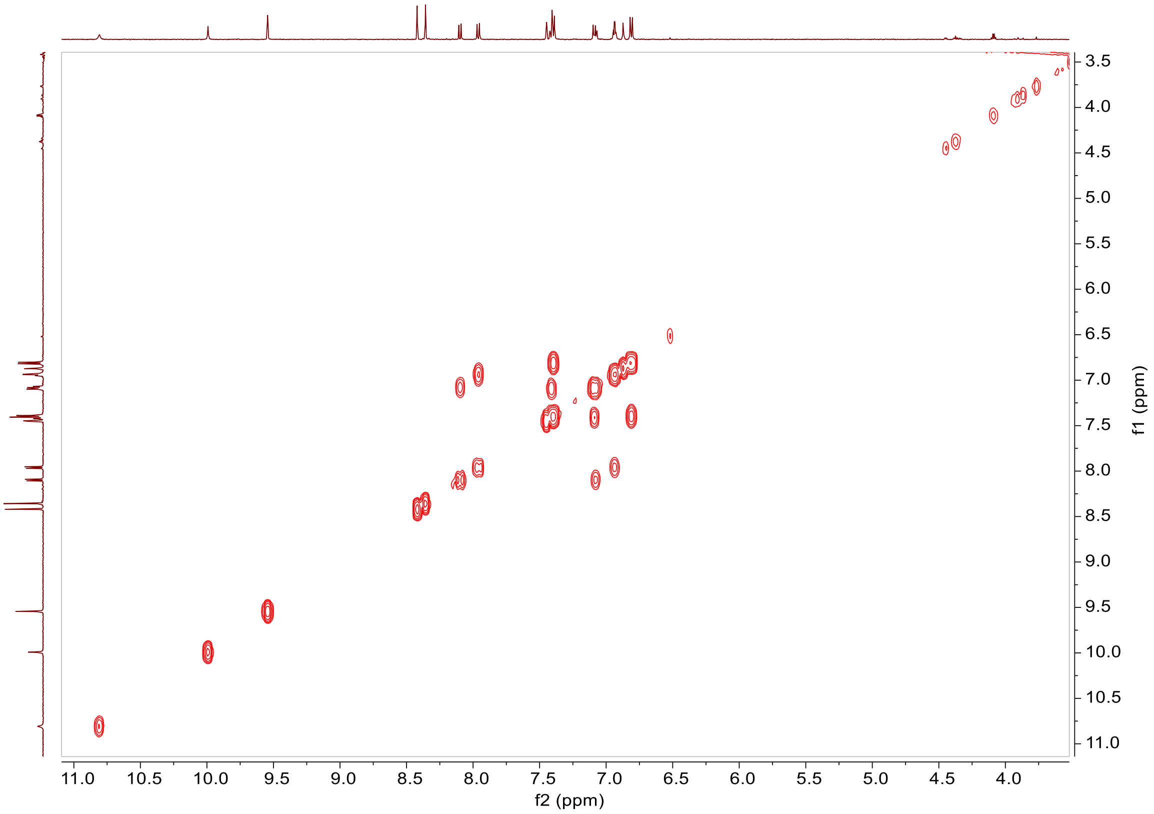

Figure S32. ^{1}H,^{1}H-COSY spectrum (500 MHz, DMSO-d_6) of **3**.

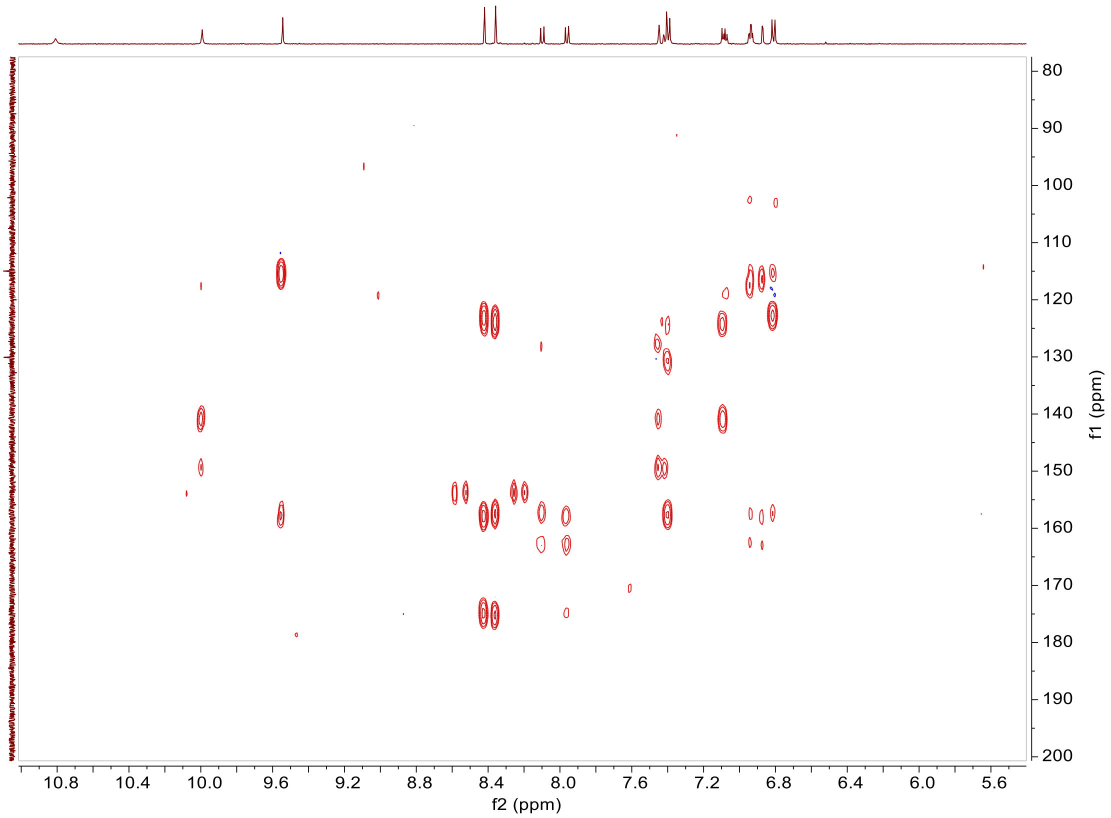

Figure S33. HMBC spectrum (600 MHz, DMSO-d_6) of **3**.

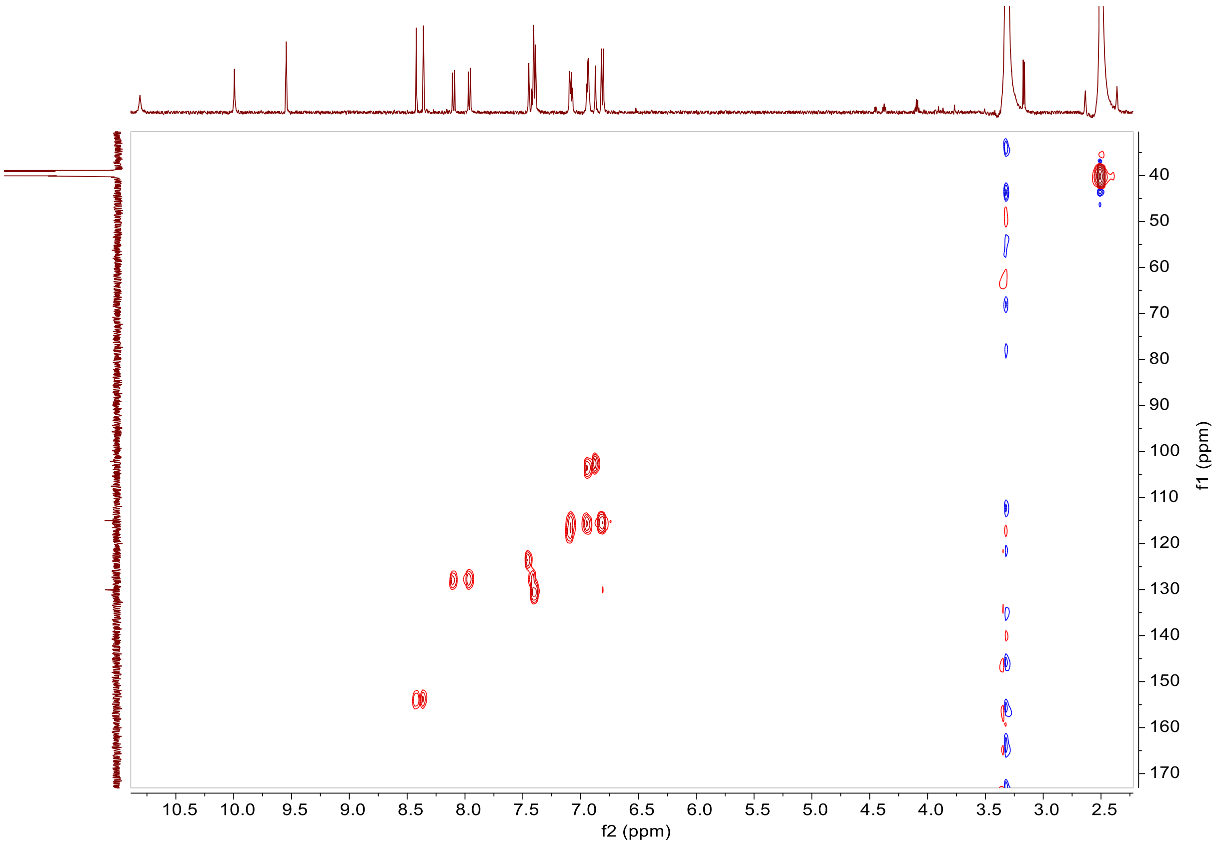

Figure S34. HSQC spectrum (600 MHz, DMSO-d_6) of **3**.

Determination of stereochemistry

Notes: Compound **1** was determined as a mixture of two atropisomers with a ratio of 3.3:1 by chiral separation (Figure S36a). According to its experimental and calculated ECD spectra, the major isomer was assigned as (*M*)-enantiomer (Figures S37 and S38). For compound **2**, only one peak was detected on chiral column, and no significant CD signal was observed. In consideration of the moiety similarity between **2** and an achiral biflavone amentoflavone (Figure S36c), this compound was favored as an achiral dimer.

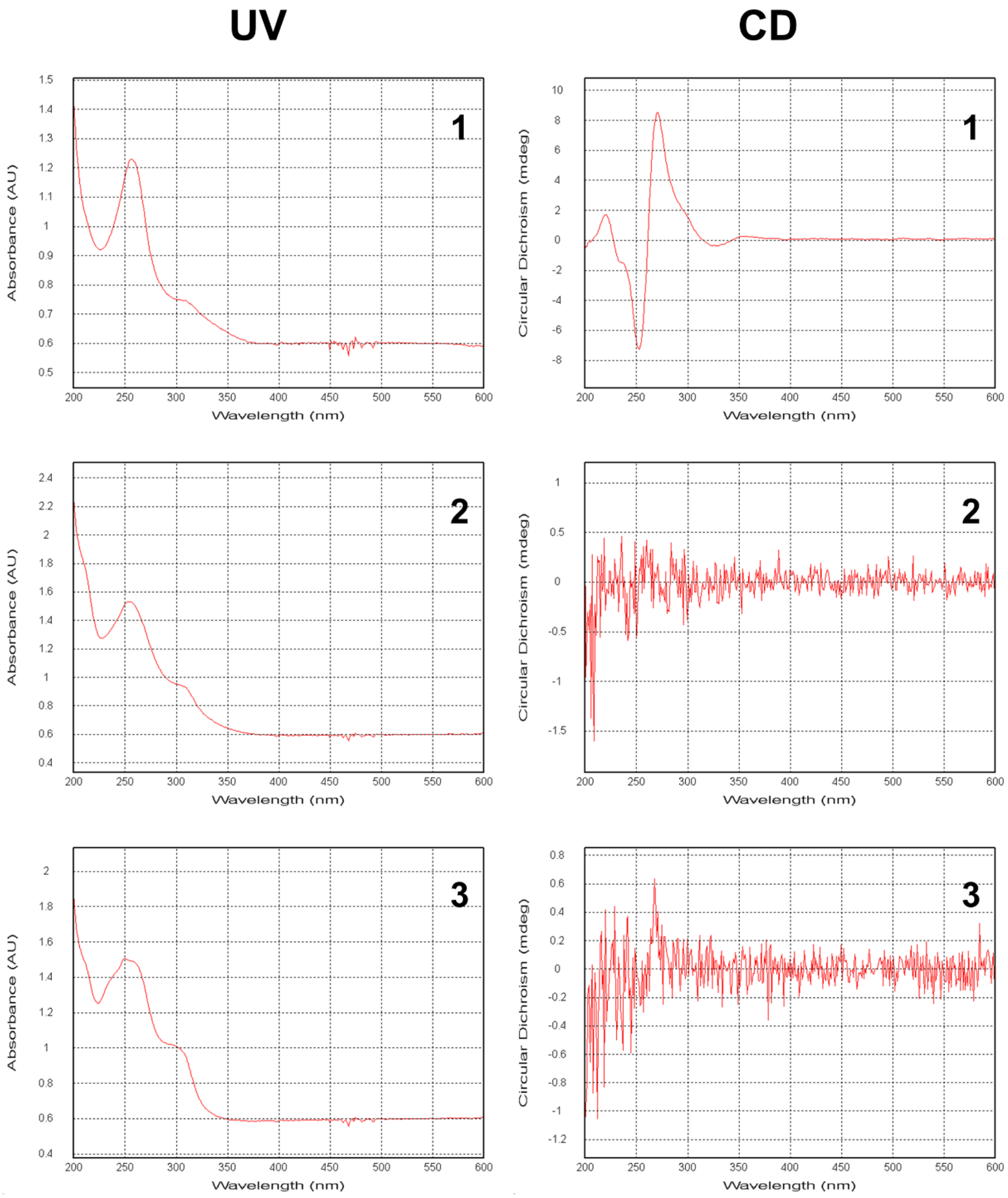

Figure S35. UV and CD spectra of **1**–**3**.

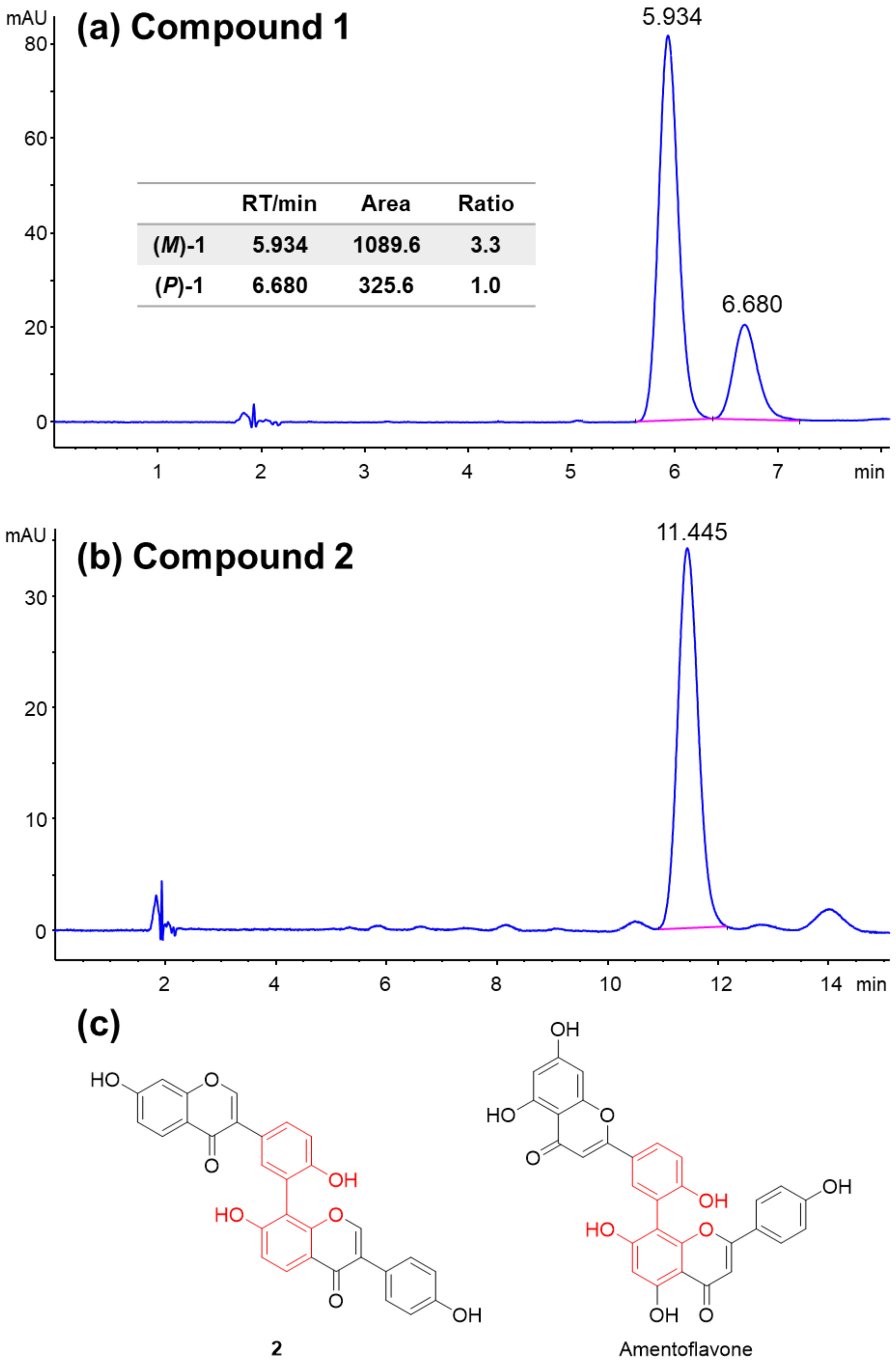

Figure S36. The HPLC analyses of **1** (a) and **2** (b) using chiral column (analytical method E, 254 nm). The assignment of each peak was confirmed by MSD detector. For example, the small peaks in (b) were excluded as isomers of compound **2** because of the absence of the ion with *m/z* 507 (ESI+). (c) The moiety comparison between **2** and an achiral biflavone amentoflavone [16].

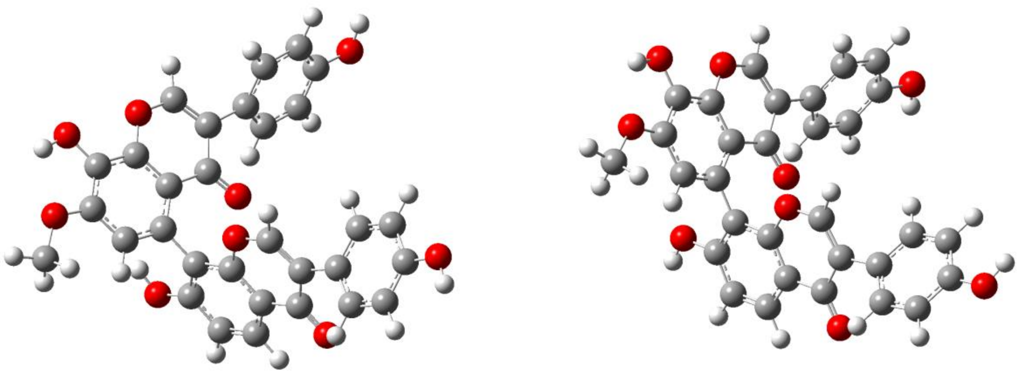

Figure S37. B3LYP/6-31+G (d,p) optimized low-energy conformers of (*M*)-**1a** (left) and (*M*)-**1b** (right).

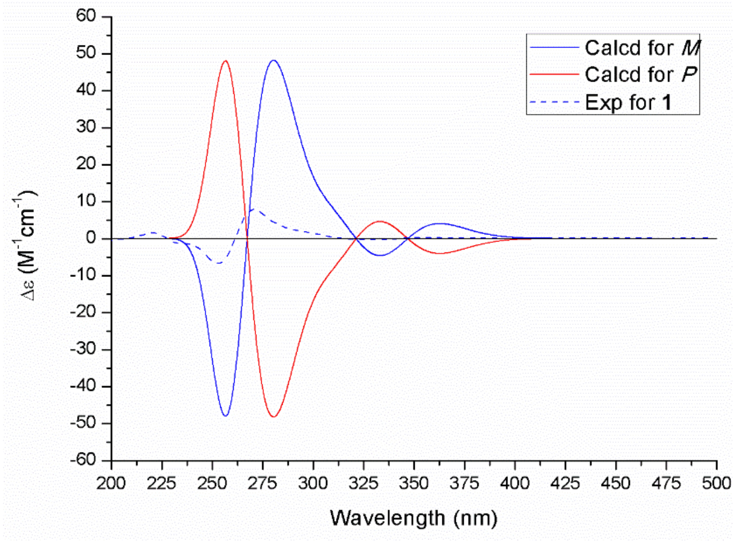

Figure S38. Experimental and calculated ECD spectra of **1**.

5. References

1. Wei, J.; Zhang, X.-Y.; Deng, S.; Cao, L.; Xue, Q.-H.; Gao, J.-M. *Nat. Prod. Res.* **2017**, *31*, 2062-2066.
2. Shaaban, K. A.; Shepherd, M. D.; Ahmed, T. A.; Nybo, S. E.; Leggas, M.; Rohr, J. *J. Antibiot.* **2012**, *65*, 615-622.
3. Funa, N.; Funabashi, M.; Ohnishi, Y.; Horinouchi, S. *J. Bacteriol.* **2005**, *187*, 8149-8155.
4. Zhao, B.; Lamb, D. C.; Lei, L.; Kelly, S. L.; Yuan, H.; Hachey, D. L.; Waterman, M. R. *Biochemistry* **2007**, *46*, 8725-8733.
5. Zhao, B.; Guengerich, F. P.; Voehler, M.; Waterman, M. R. *J. Biol. Chem.* **2005**, *280*, 42188-42197.
6. Lim, Y.-R.; Han, S.; Kim, J.-H.; Park, H.-G.; Lee, G.-Y.; Le, T.-K.; Yun, C.-H.; Kim, D. *Biomol. Ther.* **2017**, *25*, 171.
7. Präg, A.; Grüning, B. r. A.; Häckh, M.; Lüdeke, S.; Wilde, M.; Luzhetskyy, A.; Richter, M.; Luzhetska, M.; Günther, S.; Müller, M. *J. Am. Chem. Soc.* **2014**, *136*, 6195-6198.
8. Ma, J.; Wang, Z.; Huang, H.; Luo, M.; Zuo, D.; Wang, B.; Sun, A.; Cheng, Y. Q.; Zhang, C.; Ju, J. *Angew. Chem. Int. Ed.* **2011**, *50*, 7797-7802.
9. Guo, Z.; Li, P.; Chen, G.; Li, C.; Cao, Z.; Zhang, Y.; Ren, J.; Xiang, H.; Lin, S.; Ju, J.; Chen Y. *J. Am. Chem. Soc.* **2018**, *140*, 18009-18015.
10. Li, C.; Hu, Y.; Wu, X.; Stumpf, S. D.; Qi, Y.; D'Alessandro, J. M.; Nepal, K. K.; Sarotti, A. M.; Cao, S.; Blodgett, J. A. *Proc. Natl. Acad. Sci. U. S. A.* **2022**, *119*, e2117941119.
11. Agarwal, V.; El Gamal, A. A.; Yamanaka, K.; Poth, D.; Kersten, R. D.; Schorn, M.; Allen, E. E.; Moore, B. S. *Nat. Chem. Biol.* **2014**, *10*, 640-647.
12. Agarwal, V.; Moore, B. S. *ACS Chem. Biol.* **2014**, *9*, 1980-1984.
13. Sanchez, L. M. Gene deletion studies in analysis of the role of cytochrome P450 in flaviolin metabolism in Streptomyces coelicolor. Master Thesis, Swansea University, U. K., 2009.
14. Kuhn, F.; Oehme, M.; Romero, F.; Abou-Mansour, E.; Tabacchi, R. *Rapid Commun. Mass Spectrom.* **2003**, *17*, 1941-1949.
15. Nakata, R.; Yoshinaga, N.; Teraishi, M.; Okumoto, Y.; Huffaker, A.; Schmelz, E. A.; Mori, N. *Biosci. Biotechnol. Biochem.* **2018**, *82*, 1309-1315.
16. Pan, X.; Tan, N.; Zeng, G.; Zhang Y.; Jia, R. *Bioorg. Med. Chem.* **2005**, *13*, 5819-5825.
